# The Role of Kv7/M Potassium Channels in Controlling Ectopic Firing in Nociceptors

**DOI:** 10.3389/fnmol.2017.00181

**Published:** 2017-06-13

**Authors:** Omer Barkai, Robert H. Goldstein, Yaki Caspi, Ben Katz, Shaya Lev, Alexander M. Binshtok

**Affiliations:** ^1^Department of Medical Neurobiology, Institute for Medical Research Israel-Canada, Hadassah School of Medicine, The Hebrew University-Hadassah School of MedicineJerusalem, Israel; ^2^The Edmond and Lily Safra Center for Brain Sciences, The Hebrew University of JerusalemJerusalem, Israel

**Keywords:** M-current, spontaneous firing, ectopic activity, nociceptors, nociceptive terminals

## Abstract

Peripheral nociceptive neurons encode and convey injury-inducing stimuli toward the central nervous system. In normal conditions, tight control of nociceptive resting potential prevents their spontaneous activation. However, in many pathological conditions the control of membrane potential is disrupted, leading to ectopic, stimulus-unrelated firing of nociceptive neurons, which is correlated to spontaneous pain. We have investigated the role of K_V_7/M channels in stabilizing membrane potential and impeding spontaneous firing of nociceptive neurons. These channels generate low voltage-activating, noninactivating M-type K^+^ currents (M-current, *I*_*M*_), which control neuronal excitability. Using perforated-patch recordings from cultured, rat nociceptor-like dorsal root ganglion neurons, we show that inhibition of M-current leads to depolarization of nociceptive neurons and generation of repetitive firing. To assess to what extent the M-current, acting at the nociceptive terminals, is able to stabilize terminals' membrane potential, thus preventing their ectopic activation, in normal and pathological conditions, we built a multi-compartment computational model of a pseudo-unipolar unmyelinated nociceptive neuron with a realistic terminal tree. The modeled terminal tree was based on the *in vivo* structure of nociceptive peripheral terminal, which we assessed by *in vivo* multiphoton imaging of GFP-expressing nociceptive neuronal terminals innervating mice hind paw. By modifying the conductance of the K_V_7/M channels at the modeled terminal tree (terminal g_K_V_7/M_) we have found that 40% of the terminal g_K_V_7/M_ conductance is sufficient to prevent spontaneous firing, while ~75% of terminal g_K_V_7/M_ is sufficient to inhibit stimulus induced activation of nociceptive neurons. Moreover, we showed that terminal M-current reduces susceptibility of nociceptive neurons to a small fluctuations of membrane potentials. Furthermore, we simulated how the interaction between terminal persistent sodium current and M-current affects the excitability of the neurons. We demonstrated that terminal M-current in nociceptive neurons impeded spontaneous firing even when terminal Na_(V)_1.9 channels conductance was substantially increased. On the other hand, when terminal g_K_V_7/M_ was decreased, nociceptive neurons fire spontaneously after slight increase in terminal Na_(V)_1.9 conductance. Our results emphasize the pivotal role of M-current in stabilizing membrane potential and hereby in controlling nociceptive spontaneous firing, in normal and pathological conditions.

## Introduction

Primary sensory nociceptive neurons, which signals the CNS about the presence of noxious stimuli are mostly quiescent in the absence of injury-mediating stimuli (Reeh, 1986; Amir et al., 2002; Gudes et al., 2015; Emery et al., 2016). In models of inflammation or nerve injury, the stability of nociceptive membrane resting potential is dysregulated, leading to spontaneous or ectopic activity i.e., the activation of nociceptive fibers in the absence of noxious stimuli (Amir et al., 1999; Wu et al., 2001; Djouhri et al., 2006, 2012; Bernal et al., 2016). This ectopic activity is correlated to spontaneous pain (Djouhri et al., 2006; Kleggetveit et al., 2012; Serra et al., 2012). A particular class of potassium (K^+^) current, namely, K_v_7/M-current (*I*_*M*_) due to its unique biophysical properties can serve as a stabilizer of the resting potential, a kind of intrinsic “voltage clamp” mechanism (Liu et al., 2010; Passmore et al., 2012), which dampens depolarizatory deviations hence preventing ectopic firing and therefore spontaneous pain. *I*_*M*_ is generated by heteromeric K_v_7.2/3 (KCNQ2/3) channels (Brown and Passmore, 2009), which are densely expressed at the sites of spike generation e.g., axon initial segment of central neurons (Pan et al., 2006) and terminals of peripheral nociceptive neurons (Passmore et al., 2012). These low voltage-activating (around −60 mV), non-inactivating channels underlie the slow activating and prolonged outward current, which opposes membrane depolarization (Brown and Passmore, 2009). Moreover, K_v_7/M channel's activity is positively regulated by plasma membrane PtdIns(4,5)P levels (Suh and Hille, 2002; Telezhkin et al., 2012). Thus, receptors which activate the phosphoinositide lipid signaling cascade regulate *I*_*M*_ (Yu, 1995; Selyanko and Brown, 1996; Cruzblanca et al., 1998; Wen and Levitan, 2002; Gamper and Shapiro, 2003; Linley et al., 2008). Altogether, these properties position *I*_*M*_ suitable for controlling the resting potential, preventing ectopic firing in the absence of noxious stimuli, while allowing a shift to a more excitable states by receptor-mediated *I*_*M*_ inhibition. Indeed, ever since it was discovered nearly 40 year ago (Brown and Adams, 1980) *I*_*M*_ perturbations were strongly implicated in neuronal hyperexcitability underlying epilepsy and ALS (Yue and Yaari, 2004, 2006; Gu et al., 2005; Wainger et al., 2014), neuroinflammation (Tzour et al., 2016) and inflammatory, cancer and neuropathic pain (Linley et al., 2008; Liu et al., 2010; Roza et al., 2011; Zheng et al., 2013, 2015). In this context, we asked if *I*_*M*_ in nociceptive neurons, is sufficient to maintain resting membrane potential and hence prevent spontaneous activity. In central neurons, application of the selective *I*_*M*_ blocker, XE991 (Wang et al., 1998), or the activation of metabotropic glutamate receptors were shown to induce spontaneous firing (Shah et al., 2008; Lombardo and Harrington, 2016; Tzour et al., 2016). In peripheral nociceptive neurons, inhibition of *I*_*M*_ by XE991 or linopridine, another *I*_*M*_ blocker (Aiken et al., 1995) increased membrane excitability and induced membrane depolarization, but failed to induce spontaneous firing (Passmore et al., 2003; Linley et al., 2008; Liu et al., 2010). On the other hand, injection of XE991 *in vivo* to the hind paw led to prominent nocifencive behavior (Linley et al., 2012) and inhibition of *I*_*M*_ in cutaneous sensory endings in *ex vivo* skin-nerve preparation induced ectopic activity in Aδ but not in C-fibers (Passmore et al., 2012).

Here we show that inhibition of *I*_*M*_ by focal puff-application of low concentration of XE991 (either 3 or 10 μM) induces membrane depolarization followed by high frequency action potential firing in acutely dissociate rat nociceptor-like dorsal root ganglion (DRG) neurons. Using a multi-compartment computational model of a nociceptive neuron we demonstrate that *I*_*M*_, acting at nociceptive terminals is sufficient to prevent spontaneous activity of nociceptive neurons. Furthermore, *I*_*M*_ provide a “safety zone,” such that substantial changes in persistent sodium current-mediated depolarizing conductances are required to induce spontaneous firing. Decrease in terminal *I*_*M*_ induces spontaneous activation of nociceptive neurons after a small increase in persistent sodium current-mediated conductances, emphasizing the pivotal role of *I*_*M*_ in controlling nociceptive excitability.

## Materials and methods

### Ethical approval

All animal procedures were approved by the Ethics Committee of the Hebrew University (Ethic number MD-15-14274-1).

### Rat lumbar DRG cell culture preparation

Neurons were isolated from lumbar dorsal ganglions of 6–9 week old Sprague Dawley male rat as previously described (Binshtok et al., 2008; Nita et al., 2016). In short: lumbar DRGs (L1-L6) were removed and placed into DMEM with 1% penicillin-streptomycin, then digested in 5 mg ml^−1^ collagenase, 1 mg ml^−1^ Dispase II (Roche) and 0.25% trypsin, followed by addition of 0.25% trypsin inhibitor. Cells were triturated in the presence of DNase I (250 U) and centrifuged through 15% BSA. The cell pellet was re-suspended in 1 ml Neurobasal media, containing B27 supplement (Invitrogen), penicillin and streptomycin, 10 mM AraC, 2.5S NGF (100 ng ml^−1^, Promega) and GDNF (2 ng ml^−1^). Cells were plated onto poly-D-lysine (100 μg ml^−1^) and laminin (1 mg ml^−1^) coated 35 mm tissue culture dishes (Corning) at ~10 K cells per well, at 37°C with 5% carbon dioxide. Unless otherwise stated the materials were purchased from Sigma.

### General electrophysiology

**Figure 1:**
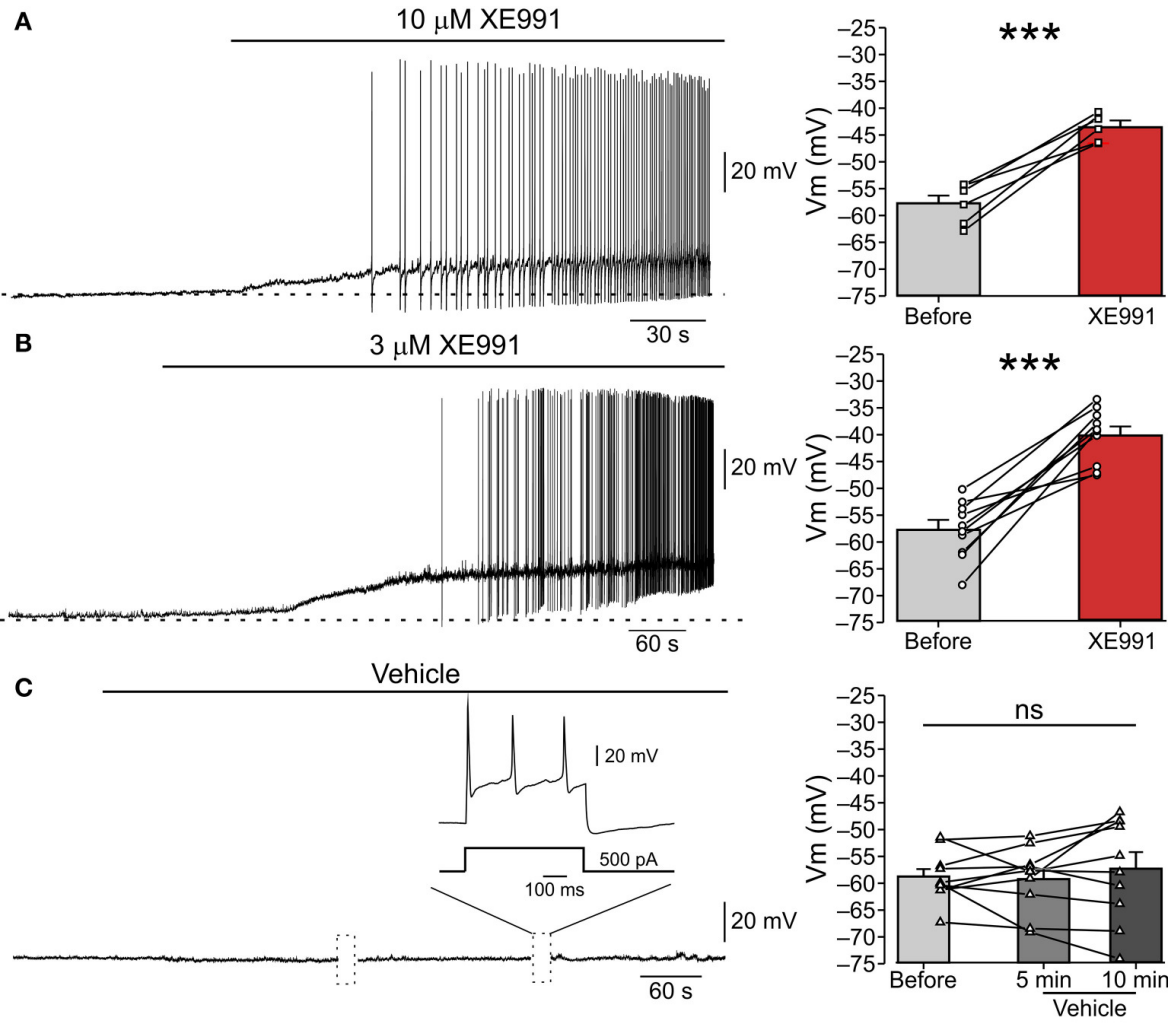
XE991 leads to spontaneous firing of nociceptor-like DRG neurons. **(A)** *Left*, Typical responses of nociceptor-like small (25 μm) DRG neurons to focal puff application of 10 μM XE991 (representative of 5 out of 6 experiments). Current clamp perforated-patch recordings performed from acutely dissociated DRG neurons perfused with extracellular solution. Note, membrane depolarization and onset of spike discharges shortly after application of XE991. Dashed line indicate resting potentials before drug application (−58 mV). *Right*, mean ± SEM (bar graphs) and individual neurons' changes in membrane potential before (*gray*) and after application of XE991 (*red*, measured before generation of first action potential). ^***^p < 0.001, paired Student *t*-test, *n* = 6. Note that XE991-induced depolarization in all recorded neurons. **(B)** Same as in **(A)**, but 3 μM XE991 was puff-applied on the neurons. Dashed line indicate resting potential before drug application (−59 mV). *Left*, representative of 10/11 neurons. ^***^p < 0.001, paired Student *t*-test, *n* = 11. Note that 3 μM XE991 induced depolarization in all recorded neurons. **(C)** Control experiment. 10 min long puff application of vehicle onto nociceptor-like DRG neuron. Note, no spontaneous depolarization developed during this period, yet the cell fired normally upon injection of depolarizing current pulses (*inset*; representative of 9 experiments). Dotted boxes show time breaks in free-run recordings when current protocols where applied. *Right*, same as in **(A,B)**, but measured 5 min and 10 min after puff application of vehicle; ns—not significant, one-way ANOVA, *n* = 9.

**Figure 2:**
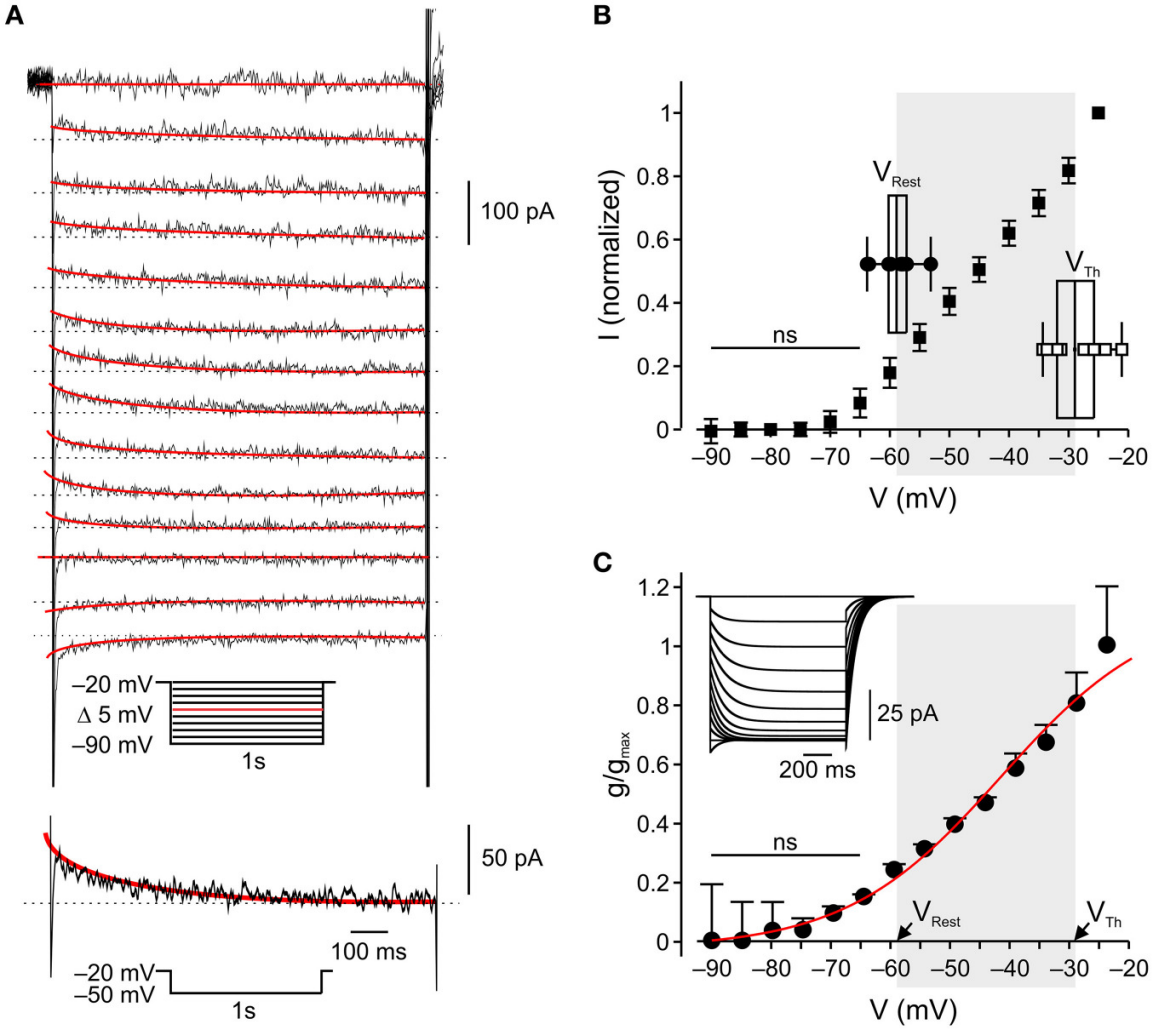
In nociceptor-like DRG neurons *I*_*M*_ is outward at resting-to-threshold potential range **(A)**. *Upper*, Typical voltage-clamp perforated patch recordings of *I*_*M*_ from a nociceptor-like DRG neuron (see Methods, representative of 18/18 neurons). A family of currents was evoked by a series of 1 s, 5 mV hyperpolarizing voltage steps from a holding potential of −20 mV (voltage protocol is shown in *inset*). The peak current response obtained by stepping to −50 mV is shown at the bottom. The *I*_*M*_ relaxation was fitted with a bi-exponential line (*red*), which was extrapolated to the beginning of the voltage step. *I*_*M*_ amplitudes were measured as the differences between the instantaneous peak currents at command onset and the steady-state currents just before command offset (*dotted line*). **(B)** Averaged leak-subtracted peak *I-V* characteristics of *I*_*M*_ recorded from nociceptor-like DRG neurons (*n* = 18), calculated as described in Methods. ns—not significant; one-way ANOVA comparison between the current values obtained at −90 mV (zero current level) to currents at other command voltages. Note, significant outward current at −60 mV. *Insets* show box charts and individual values of resting membrane potentials (V_Rest_, *n* = 10) and action potential thresholds (V_Th_, *n* = 9), obtained from current clamp perforated patch recordings (see Methods). The middle line of the box charts represents the mean; the box represents 25 ~ 75% percentiles and caps delineate range within 1.5 interquartile range. The mean (in mV) is aligned to its values on the x-axis. The shadowed area indicates the range of membrane potentials between resting potential and threshold. **(C)** Mean ± SEM of *I*_*M*_ activation curves (*g*/*g*_max_) fitted using the Boltzmann equation (see Methods) shows the onset of activation at −60 mV (ns—not significant; one-way ANOVA comparison between the conductance values obtained at −90 mV (zero conductance level) to conductances at other command voltages) and V_1/2_ at −42 mV. The shadowed area indicates the range of membrane potentials between resting potential (mean, V_Rest_) and threshold (mean, V_Th_) taken from **(B)**, *insets*.

**Figure 3:**
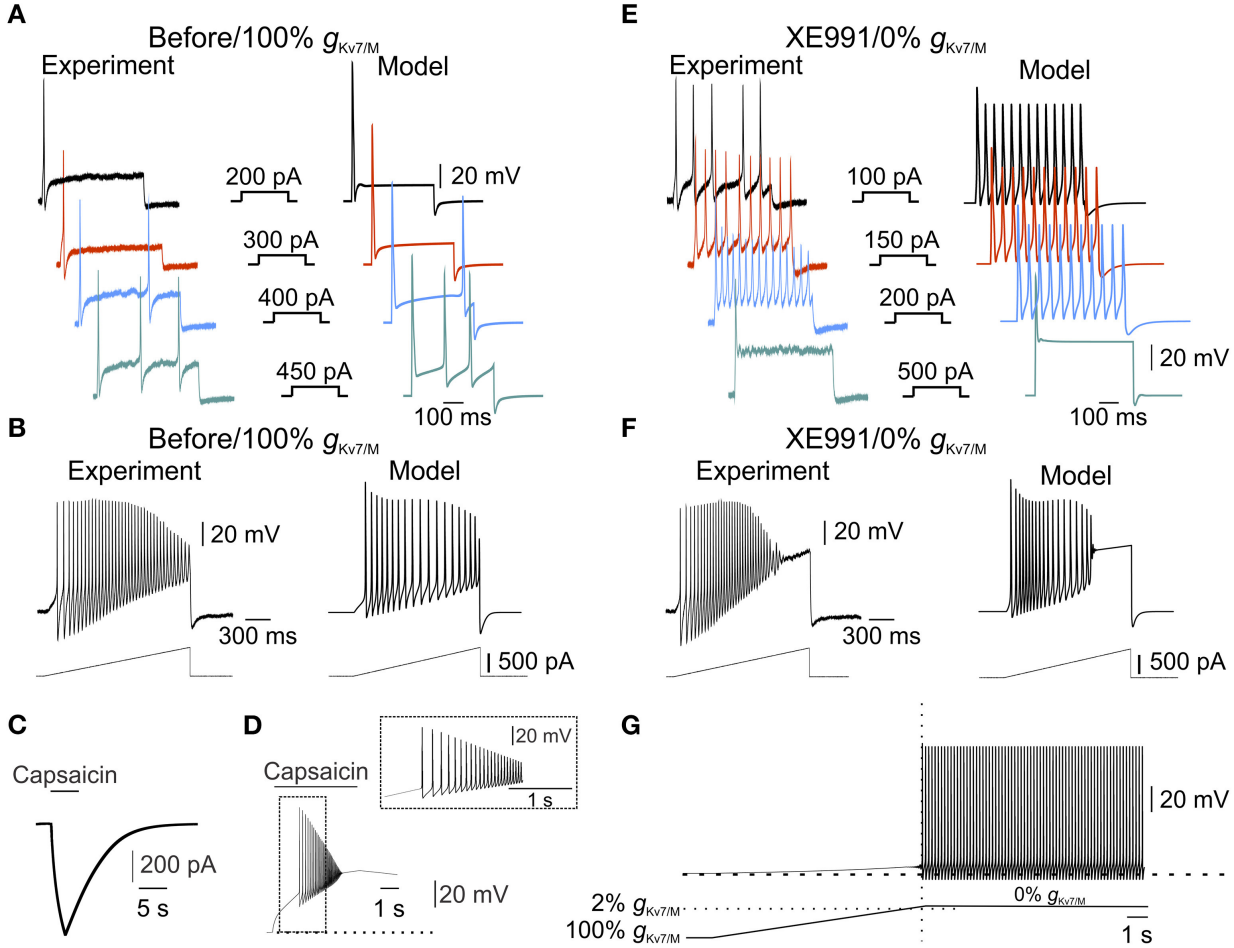
*I*_*M*_ is sufficient to prevent spontaneous firing. **(A)** Typical voltage responses to increasing current steps (showed in the *middle panel*) recorded from nociceptor-like cultured DRG neuron using perforated-patch, in current clamp mode (*left*) or from a single-compartment model (*right*, see Methods). Recordings were performed on the same DRG neuron before and after application of XE991 (shown in **(E)**, *left*). Traces from the model were obtained using the model with intact *g*_Kv7/M_. Note the similarity between the simulated to experimental responses. **(B)** Same as in **(A)**, but showing responses to 1.5 s depolarizing current ramps (250 pA/s, showed *below*) recorded from nociceptor-like cultured DRG neuron using perforated patch in current clamp mode (*left*) or from the single-compartment model (*right*). Recordings were performed on the same DRG neuron before and after application of XE991 (shown in **(F)**, *left*). Note the similarity between the simulated and experimental responses. **(C,D)** Validation of the capsaicin-like stimulation on a single compartmental model. **(C)** Current responses evoked by capsaicin-like stimulation (5 s, 1 μM, see Methods) when applied on a single-compartment model. **(D)**. Capsaicin-like stimulation evokes a barrage of action potentials in the simulated neuron. Dotted line indicates the membrane potentials before the stimulation (−57.85 mV). **(E,F)** Voltage responses to increasing current steps (**E**, showed in the *middle panel*) or current ramps (**F**, showed below) recorded from the same neuron shown in **(A,B)**, 10 min after application of 3 μM XE991 (*left*) or from the simulated neuron with *g*_Kv7/M_ = 0 (*right*). All measurements described in panels **(E,F)** were performed at the native resting potential, adjusted after XE991 application or when *g*_Kv7/M_ = 0, by injecting appropriate repolarizing currents. Note that inhibition of *I*_*M*_ leads to increase in neuronal excitability which was well reflected in the simulated neuron. **(G)** Free run recording of membrane potential in a simulated single-compartment modeled neuron during a gradual decrease in *g*_Kv7/M_ (the rate of decrease in *g*_Kv7/M_ shown below, see Methods). Dashed lines indicate resting potential before changes in *g*_Kv7/M_ (−57.85 mV). The vertical dotted line indicates time of first action potential. Horizontal dashed line indicates the level of *g*_Kv7/M_ at which the first action potential occurred. Note that in the simulated neuron, decrease in *g*_Kv7/M_ lead to slow depolarization followed by spontaneous firing, similar to the experimental results obtained after application of 3 and 10 μM XE991 on nociceptor-like DRG neurons (Figure 1).

Recordings were performed from small (~25 μm), capsaicin-sensitive (*not shown*) dissociated rat DRG neurons, up to 24 h after culturing. These neurons have been described in the literature to be nociceptive (Cardenas et al., 1995). Cell diameter was measured using Nikon Elements AI software (Nikon), from images acquired by a CCD camera (Q-Imaging). In experiments studying the role of *I*_*M*_ in nociceptor-like DRG neurons (Figures 1, 2, 3A,B,E,F, Supplementary Figure 1), whole-cell membrane currents and voltages were recorded using a nystatin-based perforated patch technique (Horn and Marty, 1988) in voltage clamp and fast current-clamp modes, respectively, using a Multiclamp 700B amplifier (Molecular Devices), at room temperature (24 ± 2°C). All other experiments (Figures 3C,D, Supplementary Figure 2) were performed using voltage and current clamp in whole cell configuration. In all experiments, only the cells that showed less than 10% change in access resistance during the entire recording period were analyzed. Data were sampled at 50 kHz and were low-pass filtered at 20 kHz (-3 dB, 8 pole Bessel filter). Patch pipettes (3–5 MΩ) were pulled from borosilicate glass capillaries (1.5 mm/1.1 mm OD/ID, Sutter Instrument Co., Novato, CA, USA) on a P-1000 puller (Sutter Instrument Co.) and fire-polished (LWScientific). Access resistance was in the range of 4–8 MΩ. For voltage-clamp recordings, capacitive currents were minimized and series resistance was compensated by about 80%. Command voltage and current protocols were generated with a Digidata 1,440 A A/D interface (Molecular Devices). Data were digitized using pCLAMP 10.3 (Molecular Devices). Data averaging and peak detection were performed using Clampfit 10.3 software. Data were fitted using Origin 8 (OriginLab).

Nystatin-based pipette solution (290 mOsm) for perforated patch recordings was freshly prepared in the dark every 2–3 h and contained (in mM): 140 KCl, 1.6 MgCl_2_, 2 EGTA, 10 HEPES, 2.5 Mg-ATP, 0.5 Na-GTP (pH = 7.4).

Nystatin (Sigma-Aldrich) was dissolved in DMSO (Sigma-Aldrich) to obtain a 50 mg ml^−1^ stock solution, which, after 1 min ultra-sonication, was diluted in pipette solution to obtain a working concentration of 125 μg ml^−1^.

The intracellular solution for measuring capsaicin-induced current contained (in mM): 145 KCl, 5 NaCl, 2.5 MgCl_2_, 2 MgATP, and 0.1 EGTA (pH = 7.4).

The intracellular solution for measuring capsaicin-evoked firing contained (in mM): 140 K-Aspartate, 10 NaCl, 2 MgCl_2_, 4 MgATP, 10 HEPES (pH = 7.4).

The extracellular solution contained (in mM): 145 NaCl, 5 KCl, 1 MgCl_2_, 10 HEPES, 10 D-Glucose (pH = 7.4).

Pipette potential was zeroed before seal formation and membrane potential was corrected for liquid junction potential of −4.5 mV.

Only cells that generated at least one action potential in response to current injections were then used for capsaicin application and analysis.

### Current clamp recordings

Only neurons that demonstrated a stable resting potential and stable action potential threshold during a 5 min application of vehicle (extracellular solution) were analyzed. In some experiments we applied vehicle solution for an additional 15 min to rule out possible time-dependent changes in neuronal excitability. No changes in the neuronal excitability were observed during the period of vehicle application (*n* = 9).

The action potential thresholds and neuronal firing properties were assessed by analyzing the neuronal responses to a series of depolarizing steps (1–2 nA in increments of 0.05 nA each) and 1.5 s depolarizing current ramps (250 pA/s). Action potential threshold was obtained by analyzing phase plots (dV/dt) of first spike evoked by 400 pA, 500 ms step when plotted versus time or versus membrane voltage. The voltage of the threshold was measured using “first local minimum” of the function before the peak. The “first local minimum” was determined as the first minimal value of dV/dt after the peak, followed by an additional increase in the dV/dt, while analyzing the function from its positive peak to time “0.” The time for the first local minimum was defined as the time of threshold, and its voltage was then detected from the original trace. For the verification of threshold values, the thresholds were also determined from the response to the depolarizing ramps, as the potential at the onset of a clear sharp deviation from passive response with subsequent action potential generation.

Measurements of changes in action potential threshold and evoked firing, following application of XE991 were made at the original (control) resting membrane potential (*V*_m_), maintained constant by injecting an appropriate steady current.

### Voltage clamp recordings

To evoke *I*_*M*_ 500 ms hyperpolarizing voltage steps incrementing by −5 mV, were applied from a holding potential of −20 mV (Halliwell and Adams, 1982; Caspi et al., 2009; Tzour et al., 2016). These steps induced slow current “relaxations” after the instantaneous inward current drops (instantaneous current), representing the slow *I*_*M*_ deactivation. Current relaxations were fitted by bi-exponential curves (starting after the capacitance artifact) and were extrapolated back to the beginning of the hyperpolarizing command pulses. *I*_*M*_ amplitudes were assessed as the differences between the instantaneous peak currents at command onset and the steady-state currents just before command offset.

The *I-V* curves were calculated according to Adams et al. (Brown and Adams, 1980) and Wang and McKinnon (1995) as follows: the intersection of *I-V* curves of normalized instantaneous and steady-state currents—V_M_, obtained from currents acquired as explained above, were measured. In our conditions V_M_ was −75 mV (*not shown*). Then, the *I*_*M*_ currents, were normalized to their maximal values and plotted vs. membrane voltages (Adams et al., 1982). The resulting *I-V* curve was upward shifted to align *I*(V_M_) to zero. Leak, obtained by extrapolation of the linear portion of the *I-V* curve between −80 to −70 mV, was then subtracted according to Passmore et al. (2003).

The conductance of *I*_M_ (g_Kv7/M_) was assessed according to Adams et al. (1982). Briefly, we averaged the currents at each command-voltage from all recorded neurons. The resulting *I-V* curve was upward shifted to align *I*(V_M_) to zero. Leak, obtained by extrapolation of the linear portion of the *I-V* curve between −80 to −70 mV, was then subtracted according to Passmore et al. (2003). The current values were then divided by the driving force and normalized to the maximal value. The data were fitted by a Boltzmann curve:

$$g/g_{max}=A_{1}-A_{2}{\left(1\;+\text{ exp}(\nu \;-\;\nu_{1/2})/k_{l}\right)}^{-1}$$

where ν_1/2_ is voltage at which the half-maximal activation of the channels occurs. The activation curve was best fitted with the following parameters: ν_1/2_ = −42*mV*, *k*_*l*_ = 12, *A*_1_ = 1.13462, *A*_2_ = 1.2071.

#### Chemicals and drugs

XE991 was purchased from Tocris Bioscience. Stock solutions were prepared in 10 mM and were diluted when added to the extracellular solution.

#### Focal drug application

XE991 or vehicle (extracellular solution) were focally and constantly applied onto somata of isolated DRG neurons using pressure puffs supplied by a pneumatic picopump PV820 (World Precision Instruments, Sarasota, FL, USA), connected to a fine pipette (3–5 MΩ). The pipette was placed about 25 μm away from the recorded cell.

Capsaicin (1 μM, Sigma) was puff-applied for 5 seconds via a pipette with a resistance of 2–5 MΩ which was placed 25 μm away from the recorded cell. Immediately after the puff application, capsaicin was washed out by perfusing with extracellular solution.

#### Computational modeling

**Figure 4:**
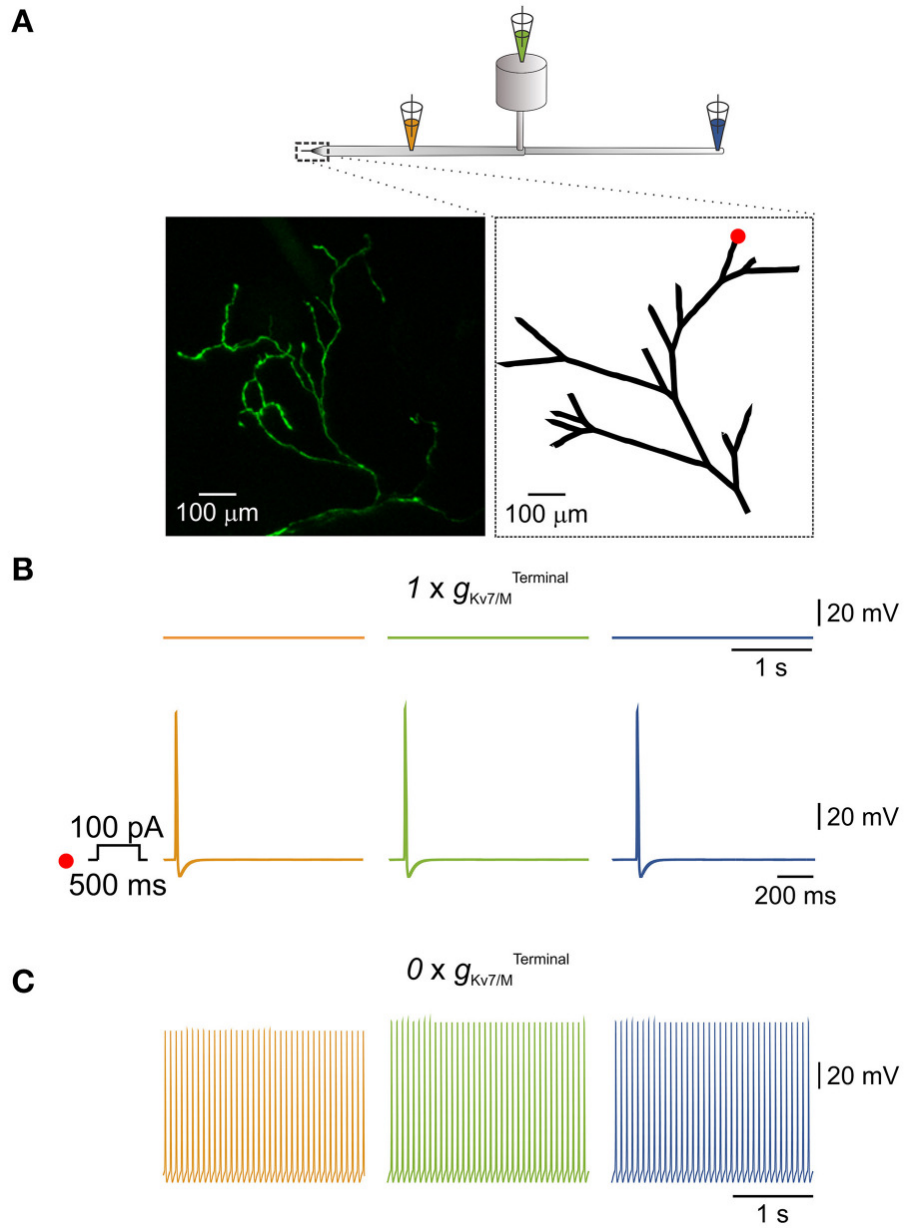
*I*_*M*_ in the terminals is sufficient to prevent spontaneous activation of a simulated multi-compartment nociceptive neuron. **(A)** Scheme depicting the general structure of the multi-compartment model. Dotted box outlines the nociceptive terminal, enlarged in the *right inset* below. Orange, green, and blue pipettes indicate the sites from which the changes in membrane potentials were recorded and correspond to: peripheral axon (*orange*), soma (*green*), and central axon (*blue*). *Insets, Left, in-vivo* multiphoton imaging of nociceptive peripheral terminals and distal axons in the mouse hind paw expressing GFP (z-stack; see Methods). *Right*, geometry of the terminal tree based on the rendering of the image shown in *left* which was used in the NEURON environment to simulate nociceptive peripheral terminals and distal axons. The red dot indicates the stimulus loci. **(B)** *Upper traces*, recordings from peripheral axon (*orange*), soma (*green*), and central axon (*blue*) when terminal K_v_7 conductance (${\text{g}}_{\text{Kv}7/\text{M}}^{\text{Terminal}}$) was intact ($1\text{ x}{\text{g}}_{\text{Kv}7/\text{M}}^{\text{Terminal}}$, see Methods). Note, no spontaneous action potential firing, yet application of 100 pA depolarizing current for 500 ms at the distal terminal branch (indicated as a red dot in **A**, *inset, right*) lead to generation of a single action potential which propagated along the neuron. **(C)** Annulling of ${\text{g}}_{\text{Kv}7/\text{M}}^{\text{Terminal}}$ ($0\text{ x }{\text{g}}_{\text{Kv}7/\text{M}}^{\text{Terminal}}$) lead to generation of spontaneous action potential firing at the distal axons (*orange*) which fully propagated along the neuron (*green and blue*).

Simulations were performed using a small-diameter non-myelinated DRG compartmental model neuron implemented and run in NEURON simulation software (Hines and Carnevale, 1997, 2000). First, the model validation was performed by simulating a single-compartment DRG soma-like 25μm X 25 μm cylinder model (Gudes et al., 2015) with a membrane capacitance of 1μF cm^−2^ and membrane resistance of 10000 Ω cm^−2^. The simulated resting potential was −57.85 mV, which fits our experimental results (−58.15±0.9 mV). The model was adjusted to reach similarity of the simulated responses compared to experimental results. Then, the single-compartment was evolved into a multi-compartment model of pseudo-unipolar unmyelinated nociceptive neuron which includes a DRG-like soma connected to a stem axon, expanding to peripheral and central neuron axons which join at a bifurcation site (T-junction, Figure 4). Simulations were performed assuming a room temperature of 25°C, the temperature at which the experimental data were collected.

#### Multi-compartment model morphology

The morphology of the cell body was based on the single compartment model. The morphology and passive properties of the T-junction and axons were based on previous studies (Du et al., 2014; Sundt et al., 2015). The terminal morphology was based on our experimental observations (see Figure 4 and Methods below) and was constructed accordingly. The terminal tree contained a total of 27 branches with variable lengths (50–300 μm) and a diameter of 0.25 μm. The membrane resistance of the nerve ending branches was 4-fold somatic membrane resistance (Vasylyev and Waxman, 2012). To achieve morphological continuity for proper electric propagation from the terminal tree to the peripheral axon the two compartments were connected by a tapered cone-like axon with a linearly changing diameter over 100μm of length.

#### Active conductances

The model includes active conductances simulating the following channels: TTX-sensitive sodium current (I_NattxS_), TTX-s sensitive persistent sodium current (I_NaP_), Nav1.9 TTX-r sodium channels (I_Nav1.9_) and Nav1.8 TTX-r sodium channels (I_Nav1.8_). All channels parameters of the sodium currents were adapted from Herzog et al. (Herzog et al., 2001) and Baker et al. (Baker, 2005). Three types of potassium channels included: (i) the delayed rectifier channel (I_KDR_) adapted from (Herzog et al., 2001); (ii) An A-type potassium channel (I_KA_) adapted from Miyasho et al. (Miyasho et al., 2001), whose activation and inactivation gates were shifted by 20 mV in hyperpolarized direction to closely resemble kinetics of DRG neurons (Qu and Caterina, 2016) and (iii) the Kv7/M channels which were adapted from Shah et al. (Shah et al., 2008) and tuned to our experimental results. The h-current (*I*_h_) was also included and taken from Shah et al. (Shah et al., 2008), the slope factor was tuned according to Komagiri and Kitamura (Komagiri and Kitamura, 2003).

For simulating the excitable properties of a single-compartment neuron we used the following fixed maximal conductance parameters:

$$\begin{array}{l} g_{\text{Nav}1.8}=0.02S/cm^{2},\;g_{\text{Nav}1.9}=0.00064S/cm^{2},\\ g_{\text{NaTTXS}}=0.0017S/cm^{2},\\ g_{\text{NaP}}=0.00005S/cm^{2},g_{\text{KDR}}=0.00083S/cm^{2},\\ g_{\text{KA}}=0.0015S/cm^{2},\\ g_{\text{Kv}7/\text{M}}=0.00034S/cm^{2},g_{\text{H}}=0.00033S/cm^{2}. \end{array}$$

Since there is no information available regarding the distribution of channels across the nociceptive axons, for maintaining simplicity, conductances were evenly distributed in all compartments.

#### Kv7/M-current potassium current model

The Kv7/M current was defined as:

$$I_{K\nu 7/M}=g_{K\nu 7/M}\cdot m\cdot (\nu -E_{K})$$

where *g*_*Kv*7/*M*_ is maximal conductance, *m* is the activation gate parameter, *v* is the membrane potential and *E*_*K*_ is the potassium reversal potential value.

To implement our experimental results, we adjusted the voltage-dependence of steady-state activation for Kv7/M current to our findings (see Figure 2). The activation curve was fitted using the Boltzmann equation to fit the activation gate parameter:

$$m=A_{1}\;-\;A_{2}\frac{1}{1\;+\;exp((\nu \;-\;\nu_{1/2})/k_{l}}$$

where ν_1/2_ is voltage at which the half-maximal activation of channels occurs. The activation curve was best fitted with the following parameters: ν_1/2_ = −42*mV*, *k*_*l*_ = 12, *A*_1_ = 1.13462, *A*_2_ = 1.2071. The time constants were taken from Shah et al. (Shah et al., 2008).

To simulate the currents inhibition by XE991 we decreased the value of g_*Kv*7/*M*_ as mentioned in different simulations in this study. In some experiments (Figure 3G) g_*Kv*7/*M*_ was decreased linearly with time according to:

$$\begin{array}{l} For\;t_{init}\leq t\leq t_{Final}\\ g_{Kv7/M}\left(t\right)=g_{Kv7/M}-g_{Kv7/M}\left(\frac{t-t_{init}}{t_{Final}-t_{init}}\right) \end{array}$$

where *t*_*init*_ is the time of the beginning of change in g_*Kv*7/*M*_; *t*_*Final*_ is a time by which the *g*_*Kv*7/*M*_ = 0. The decrease of g_KV7/M_ was modeled in a linear and fast (seconds) fashion for computational resource and simplicity reasons, it was not intended to fully replicate the time course of experimental pharmacological *I*_*M*_ blockade.

The equilibrium potentials for Na^+^ (E_Na_) and K^+^ (E_K_) were +60mV and −85mV respectively. E_Rev_ for Ih was −20 mV. The leak reversal potential was adjusted to achieve a resting potential close to our experimental results (−58.15±0.9 mV) such that the resulted resting potential of the modeled neuron was −57.85 mV. All recordings were made after letting the simulated membrane potential reach a steady-state value.

To model terminal *g*_*Kv*7/*M*_ ($g_{\text{Kv}7/\text{M}}^{\text{Terminal}})$, we used the parameters of somatic *g*_*Kv*7/*M*_ ($g_{\text{Kv}7/\text{M}}^{\text{Soma}}=0.00034s/cm^{2})$ for which the values were adjusted for the area of the cable-like terminal. This value was considered the intact $g_{\text{Kv}7/\text{M}}^{\text{Terminal}}$ and was increased or decreased by multiplying it by varying factor. The relevant figures show the multiplication factor as independent variable.

#### Capsaicin puff-like stimulus

A capsaicin-like induced current was introduced into a single simplified voltage clamp point-process with a fast exponential activation and slow exponential inactivation mimicking the experimental kinetics of puff-applied 1 μM capsaicin induced current (Nita et al., 2016):

for *t* ≤ *t*_*puff*_

$$I_{Capsaicin}\;=\;g_{Cap}\cdot \alpha \left(t-t_{onset}\right)\cdot (V-E_{TRPV1})$$

for *t*_*puff*_<*t*

$$\begin{array}{l} I_{Capsaicin}=g_{Cap}\cdot \alpha \left(t-t_{onset}\right)\cdot \beta \left(t-(t_{onset}+t_{puff})\right)\cdot (V-E_{TRPV1})\\ \alpha \left(x\right)=1-e^{-\frac{x}{t_{a}}}\\ \beta \left(x\right)=e^{-\frac{x}{\tau_{\beta }}} \end{array}$$

where *g*_*Cap*_ is the maximal conductance, α(*x*) and β(*x*) are the activation and inactivation functions with τ_α_ and τ_β_ as the activation and inactivation time constants respectively. *t*_*onset*_ and *t*_*puff*_ are the times at which the puff application simulation begins and the length of application respectively. *E*_*TRPV*1_ represents the reversal potential for TRPV1 cation channel.

The current was first tuned to fit experimental results (Supplementary Figure 2A, see also Nita et al., 2016) and then it was applied to the model neuron to verify that the induced voltage response for the experimental capsaicin-induced firing (Supplementary Figure 2A, see also Nita et al., 2016). Since our experimental recordings were obtained in DRG culture neurons which are considered to consist of only DRG soma, both adjustment and verification were performed in a soma-like model. The parameters for the capsaicin-like current were tuned to following values:

$$g_{Cap}=9\mu S/cm^{2},\tau_{\alpha }=1\cdot 10^{6}ms,\tau_{\beta }=6500ms,E_{TRPV1}=0$$

In experiments simulated voltage clamp, to avoid action potential-mediated escapes from voltage clamp, capsaicin-like currents were measured when Na(v)1.8 conductance was zeroed.

#### Noise current

The noise which was injected into whole terminal tree, was based on the Ornstein-Uhlenbeck process with mean 0, and the current was taken from Olivares et al. (Olivares et al., 2015).

$$\frac{dI_{Noise}}{dt}=\frac{-I_{Noise}\;+\;sN(t)}{\tau_{Noise}}$$

Where σ is square root of the steady state variance of the current amplitude; N - is a normally distributed random variable with zero mean and variance = 1 and τ is the steady-state correlation time length of the current (was fixed to 1 ms).

#### Terminal excitation

When simulating an electric stimulation and recording from a single terminal, a NEURON “point process” electrode was positioned, stimulus was given and data was collected at an arbitrarily chosen single terminal branch - “Terminal (Yu, 1995)”. To avoid boundary condition problems, the stimulating electrode or capsaicin puff-like process were positioned at 15% of the branch length taken from the branch's distal ending.

#### *In-vivo* sciatic viral injections

Viral injection to the sciatic nerve was performed similarly to previously described (Towne et al., 2009; Iyer et al., 2014). In short: 4-6 week old male C57/BL6 mice were anesthetized by 3% isoflurane for induction of anesthesia and kept at 1-1.5% isoflurane throughout the procedure. Mice were treated with 10 mg/kg carprofen via subcutaneous injection prior to surgery and placed on a heating pad maintained at 37 °C. After properly cleaning and disinfecting the skin of the left hind limb and lower back of mice, a 1 cm incision was made around the knee area and the sciatic nerve was exposed at the bifurcation of the Common Peroneal and Tibial nerves. A 35G beveled needle (Nanofil no. NF35BV-2, World Precision Instruments) was threaded into the Tibial branch of the nerve and 5 μl of an adeno-associated virus serotype 6 (AAV6) carrying an expression cassette for GFP under the CMV promotor (AAV6-CMV-GFP, ELSC Viral Core, The Hebrew University) was injected at a rate of 1 μl/min, using a 10 μl syringe (Hamilton Company) connected to a UltraMicroPump (UMP3) with a SYS-Micro4 Controlled WPI Syringe pump (WPI). After injection, the needle was kept inserted in the nerve for another 5 min to allow for pressure stabilization. The incision was then sutured and treated with 3% synthomycin ointment (Rekah, Israel). Mice were treated post-operatively every 12 hours for 3 days with 5 mg/kg carprofen.

#### *In-vivo* multiphoton Z-stack projection of peripheral free nerve endings innervating the skin of the mouse hind paw

Imaging was performed similarly to previously described (Yuryev and Khiroug, 2012). In short: 4 weeks post viral injection; mice were anesthetized by 3% isoflurane for induction of anesthesia and kept at 1-1.5% isoflurane throughout the experiment. Mice were placed under the microscope on a heating pad maintained at 37°C and the left hind paw was placed in between 2 glass slides with a few drips of 0.9% w/v NaCl solution between the skin of paw and glass. Hind paw and glass slides were stabilized by a Noga holding system (Noga Engineering, Ltd, Israel). Multiphoton Z-stack projection of free nerve endings in hind paw was performed using a Zeiss LSM 7 MP system mounted on a Multiphoton Axio microscope (Carl Zeiss), a Chameleon Ultra II Diod-Pumped Laser (Coherent) and Zen2010 software (Carl Zeiss). The objective used for fluorescence collection was a W Plan-Apochromat 20x/1.0 DIC CG = 0.17 M27 75 mm water immersion lens (Carl Zeiss). Laser excitation wave length was set at 900 nm and average power set to 200 mW. Emission was collected by two channel NDD PMTs, for green and red emission wave lengths. Images were acquired at a resolution of 1024 × 1024 pixels in the x, y plane (530.85x530.85 μm, 8 bit, 0.8 zoom) and z vertical steps were set at 0.5 μm (image was cropped for figure). 172 sections were acquired giving an image z projection of 86 μm. Images were further processed off-line using FIJI (ImageJ) for subtraction of red channel acquisition and linear un-mixing of the two channels to remove auto-fluorescence of surrounding skin structures.

#### Data analysis

Offline analyses were performed with pCLAMP 10.3 software (Molecular Devices), Matlab and OriginPro v8. Assessment of statistical significance of differences between means was performed with Student's paired *t* test or repeated-measures of ANOVA, as appropriate. Data are presented as means ± SEM.

## Results

### Application of the I_*M*_ inhibitor, XE991, causes repetitive firing of cultured nociceptor-like rat DRG neurons

To test if *I*_*M*_ prevents spontaneous activity of nociceptive DRG neurons we performed perforated-patch clamp recordings in current-clamp mode from acutely dissociated small (less than 25 μm in diameter), nociceptor-like DRG neurons. At resting potential (−58.15±0.9 mV, Figure 1, *right* “Before”), all neurons were quiescent (*n* = 26, Figure 1). Focal puff application of the *I*_*M*_ blocker XE991 (10 μM; applied ~2 min after the onset of recording) caused, within 3-5 min, a substantial depolarization (12.35 ± 1.8 mV; Figure 1A, *right* “XE991”) in all recorded neurons followed by repetitive firing in 83% of the neurons (5/6; Figure 1A, *left*). This excitatory effect persisted throughout drug application (1-5 min) and in most cases was also observed during washout (*not shown*).

Several recent reports demonstrated that 10 μM XE991 can also affect Kv1 and Kv2/Kv9 channels (Zhong et al., 2010) and delayed rectifier (*I*_*K*_) and A-type (*I*_A_) potassium currents (Xia et al., 2002). We therefore performed the abovementioned experiment using focal application of a lower concentration of XE991 (3 μM) and showed similar effects on nociceptor-like DRG neuronal excitability. Puff application of 3 μM XE991 lead, within 4–6 min, to 17.58 ± 2.4 mV membrane depolarization in all neurons and repetitive firing in 91% of the neurons (10/11; Figure 1B). To reassure that the observed spontaneous activity was due to XE911 mediated *I*_*M*_ inhibition and not due to mechanical interference caused by pressure during puff application, we recorded changes in nociceptor-like DRG neurons activity following puff application of vehicle (see Methods). Puff applied vehicle exerted no neuronal effects during sustained recordings (up to 15 min; *n* = 9; Figure 1C). In the latter condition the neurons displayed normal evoked firing behavior (*n* = 9; Figure 1C).

### I_*M*_ is outward at resting potential of nociceptor-like DRG neurons

The abovementioned results, showing that selective inhibition of *I*_*M*_ leads to spontaneous activity in nociceptor-like DRG neurons, indicate that *I*_*M*_ provides sufficient outward current to prevent suprathreshold neuronal depolarization in the resting-to-threshold membrane potential range. To characterize the activation properties of the K_v_7 channels underlying *I*_*M*_ in this membrane potential range, we first measured the action potential threshold of nociceptor-like DRG neurons using perforated-patch clamp recordings in the current clamp mode. We measured spike threshold by analyzing the phase plot (dV/dt) of single action potential evoked by 500 ms steps. We further verified the action potential threshold using 500 ms current ramps (see Methods). In our experimental conditions the threshold for action potential in nociceptor-like neurons was −31.1±2.2 mV (*n* = 14), which was similar to the one measured using ramps (−29.4±2.2, mV *n* = 9, see *inset* for Figure 2B).

Next we characterized the voltage dependence of *I*_*M*_ activation. We used a well-established voltage-clamp protocol in the perforated-patch whole cell configuration to isolate *I*_*M*_ and to generate *I-V* and conductance slope curves (Brown and Adams, 1980; Passmore et al., 2003; Wang and McKinnon, 1995; see Methods; Figure 2A, *inset*). In our conditions *I*_*M*_ was activated at −60 mV (*n* = 18, Figures 2B,C) similarly to what was previously shown (Passmore et al., 2003). Considering the reversal potential of *I*_*M*_ (Figure 2B), these data indicate that *I*_*M*_ is active and hyperpolarizatory at resting-to-threshold potentials in nociceptor-like DRG neurons.

Finally, we verified that *I*_*M*_ in nociceptor-like DRG neurons was attenuated by 3 μM XE991 (Supplementary Figure 1).

### I_M_ is sufficient to prevent spontaneous firing of nociceptor-like DRG neurons

Our data show that XE991 abolishes *I*_*M*_ and leads to spontaneous firing. To link between these two effects of XE991 and to show that *I*_*M*_ is sufficient for averting nociceptive ectopic activity, we built a single-compartment computational model of a nociceptor-like DRG neuron using NEURON simulating environment (Hines and Carnevale, 1997). We used our experimental data to determine the kinetic properties of the *I*_*M*_-mediated conductance and tuned the *I-V* relationship according to the steady-state *I-V* curve shown in Figure 2B. We adapted the *I*_*M*_ current model from Shah et al. (Shah et al., 2008) and adjusted its parameters to fit our findings. The properties of the resulting simulated current resembled the amplitude, kinetics and voltage dependence of *I*_*M*_ recorded from nociceptor-like DRG neurons (Figure 2C, *inset*). To simulate electrical properties of DRG neurons, we based our model on previously described models of nociceptor-like DRG neurons (Gudes et al., 2015; Herzog et al., 2001; Baker, 2005; see Methods). Under these conditions the resting potential of the simulated membrane was −57.85 mV and the threshold was −30.27 mV, which were similar to the experimental values (shown in Figure 2B, *insets*). Importantly, the simulated responses to current steps (Figure 3A) and depolarizing ramps (Figure 3B) replicated well the experimental findings. We then validated the response of the modeled soma to “natural” stimuli. To that end, we simulated a current induced by application of capsaicin, an activator of the noxious heat-sensitive TRPV1 channel, expressed by nociceptive neurons. We and others have previously demonstrated that application of capsaicin produces depolarization of nociceptive neurons, followed by a stereotypic pattern of action potential firing (Nita et al., 2016; Blair and Bean, 2003; see also Supplementary Figure 2, here). To mimic the capsaicin-induced current, we first introduced an inward current with the activation and inactivation kinetics resembling TRPV1-induced inward current evoked by short (5s) puff application of capsaicin (1 μM) into our single-compartment model (see Methods). The stimulation of the soma with a modeled 5 s application of capsaicin (capsaicin-like stimulation) resulted in an inward current and action potential firing, which resembled the experimental data (Figures 3C,D, compare to Supplementary Figure 2A,B respectively, see also Nita et al., 2016).

Next, we explored whether our model would reflect the hyperexcitable changes following inhibition of *I*_*M*._ It is widely accepted that *I*_*M*_ inhibition apart from membrane depolarization (Figure 1) leads to decrease in spike threshold and increase in firing rate (Yue and Yaari, 2004; Gu et al., 2005; Linley et al., 2008; Liu et al., 2010; Tzour et al., 2016). Similarly, in our experimental conditions inhibition of *I*_*M*_ decreased spike threshold (−34.94±4.9 mV, *n* = 8) and increased action potential firing evoked either by depolarizing steps (compare Figure 3A, *left* to Figure 3E, *left*) or ramps (compare Figure 3B, *left* to Figure 3F, *left*). To examine if our model would reflect the hyperexcitability induced by *I*_*M*_ inhibition we omitted the *I*_*M*_-like conductance (*g*_Kv7/M_) from the model without altering any of the other parameters. The negative steady state current was applied to hyperpolarize the membrane to its initial value. In these conditions, the action potential threshold was decreased to −32.82 mV. Moreover, application of depolarizing steps or ramps accurately reflected the changes we observed in our experimental conditions when 3 μM XE991 was puff applied onto the nociceptor-like DRG neurons (Figures 3E,F). Thus, our model accurately predicts excitable properties of nociceptor-like DRG neurons, under the limitations and assumptions made (see Methods).

Finally, using this model, we show that gradual attenuation of *g*_Kv7/M_ (see Methods) lead to membrane depolarization followed by spontaneous action potential firing (Figure 3G). The action potential firing was induced only when at least 98% of somatic *g*_Kv7/M_ (${\text{g}}_{\text{Kv}7/\text{M}}^{\text{Soma}})$ was blocked (Figure 3G, *bottom*). These simulated results suggest that *I*_*M*_ is sufficient to prevent spontaneous activity of nociceptor-like DRG neurons.

### I_*M*_ controls the ectopic activity of the neuron, in simulated nociceptive terminals

Nociceptive DRG somata robustly express K_v_7 channels which underlie *I*_*M*_ (Passmore et al., 2003). In addition, K_v_7 channels are present at nociceptive peripheral terminals innervating the target organs (Passmore et al., 2012). Thus *I*_*M*_, as an integral part of the terminal conductance may contribute to the terminal excitability state. Indeed, subcutaneous application of XE991 into the hind paw induced increased grooming and flinching, suggesting that inhibition of *I*_*M*_ at the terminals and distal axons leads to acute pain (Linley et al., 2008). It is therefore plausible that K_v_7 channels, which are expressed at the nociceptive terminals prevent spontaneous activation of terminal and hence the ectopic activity of nociceptive neurons. To examine whether *I*_*M*_ is sufficient to prevent spontaneous activation of nociceptive terminals we built a realistic multi-compartment model of nociceptive peripheral terminal and peripheral and central axons connected to cylindrical soma via a T-junction and stem axon (Figure 4A). To be able to closely replicate the geometry of the nociceptive terminal branches, we first assessed the structure of the nociceptive terminal tree *in-vivo* using multiphoton imaging of nociceptive terminal branches innervating the skin. To that end, we expressed a fluorescent marker in the sensory neurons innervating the mouse hind paw by infecting mice sciatic nerve unmyelinated C-fibers (Iyer et al., 2014) with an AAV6 carrying the expression cassette for GFP. The intact axons and terminals innervating the mice hind paw were visualized *in-vivo* and reconstructed in 3D, using multiphoton microscopy, two weeks after sciatic injection, as exemplified in Figure 4A, inset *right*. The 2D render of this 3D reconstruction was then used as a base for building a geometrical modeled structure of the terminal tree (Figure 4A, inset *left*).

We based some of the electrical parameter values of the model (see Methods) on data available from the literature (Pristera et al., 2012; Vasylyev and Waxman, 2012; Feng et al., 2015; Sundt et al., 2015), while some were obtained by data extrapolation. The model incorporates a repertoire of voltage-gated Na^+^, K^+^ conductances homogeneously distributed along the axons and the terminals and tuned to resemble experimental values (see Methods). To model terminal *g*_Kv7/M_ ($g_{\text{Kv}7/\text{M}}^{\text{Terminal}})$, we used the parameters of somatic *g*_*Kv*7/*M*_ ($g_{\text{Kv}7/\text{M}}^{\text{Soma}})$ adjusted for the area of the cable-like terminal (see Methods). When the $g_{\text{Kv}7/\text{M}}^{\text{Terminal}}$ was intact ($1\times g_{\text{Kv}7/\text{M}}^{\text{Terminal}}$, see Methods) the simulated neuron was quiescent (Figure 4B, *upper traces*). In the latter condition, stimulation of one of the terminal branches (Figure 4A, inset *right*, red dot) by a 500 ms square pulse of 100 pA current, elicited activation of the distal axon which propagated along the soma and reached the central terminal (Figure 4B, *lower traces*). When $g_{\text{Kv}7/\text{M}}^{\text{Terminal}}$ was zeroed ($0\times g_{\text{Kv}7/\text{M}}^{\text{Terminal}})$ in the whole terminal tree (shown in Figure 4A, inset, *right*) a spontaneous firing with a rate of 13 Hz (the maximal firing rate in the model) was observed, which started at the peripheral axon and propagated throughout the neuron (Figure 4C). These data suggest that *I*_*M*_ acting at the nociceptive terminals is sufficient to prevent spontaneous activation and therefore ectopic activity of nociceptive neurons.

**Figure 5:**
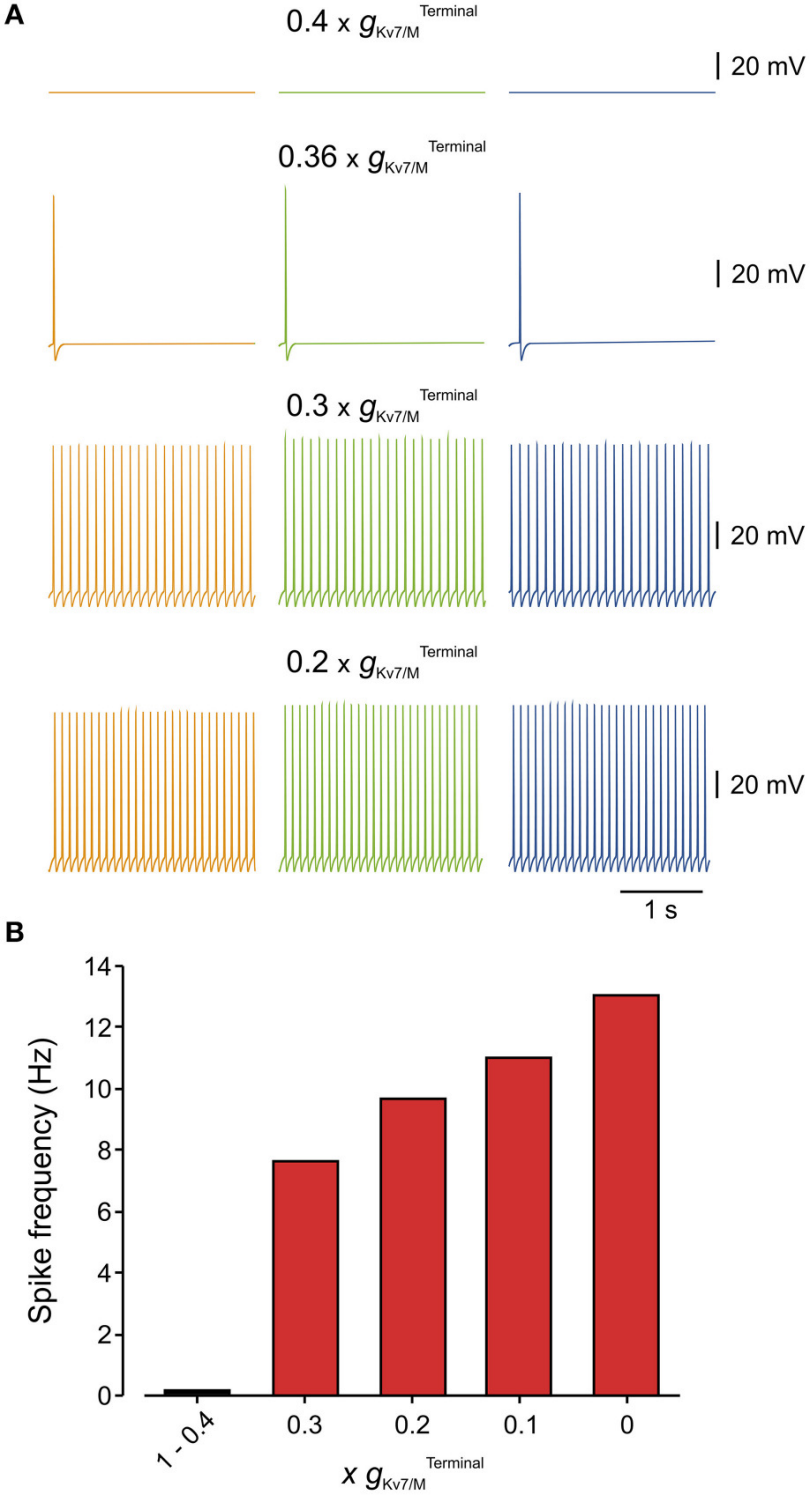
Only 40% of terminal *g*_Kv7/M_ is sufficient to prevent spontaneous firing of the modeled multi-compartment nociceptive neurons. **(A)** Simulated recordings of changes in membrane potentials obtained from a multi-compartment model at peripheral axon (*orange*), soma (*green*), and central axon (*blue*, see Figure 4). Monitoring of spontaneous firing was performed when different levels of $g_{\text{Kv}7/\text{M}}^{\text{Terminal}}$ were added to the model. Note that when 60% of $g_{\text{Kv}7/\text{M}}^{\text{Terminal}}$ was blocked ($0.4\times g_{\text{Kv}7/\text{M}}^{\text{Terminal}}$) the neuron was still silent. Inhibition of 64% of $g_{\text{Kv}7/\text{M}}^{\text{Terminal}}$ ($0.36\times \;g_{\text{Kv}7/\text{M}}^{\text{Terminal}}$) lead to spontaneous firing of 2 action potentials. Note also that the frequency of spontaneous firing increased with the decrease in $g_{\text{Kv}7/\text{M}.}^{\text{Terminal}}$ **(B)** Bar graph plotting the spike count at different levels of ${\text{g}}_{\text{Kv}7/\text{M}}^{\text{Terminal}}$ inhibition (shown as a factor by which $g_{\text{Kv}7/\text{M}}^{\text{Terminal}}$ was multiplied) measured from the central axon of the multi-compartment model (*blue electrode* in Figure 4). At $1\times g_{\text{Kv}7/\text{M}}^{\text{Terminal}}$ (no inhibition) till $0.4\times g_{\text{Kv}7/\text{M}}^{\text{Terminal}}$ (60% inhibition) no spontaneous firing was observed, thus these values were combined into one bar.

We next estimated, using our multi-compartment model, what is the value of $g_{\text{Kv}7/\text{M}}^{\text{Terminal}}$ which is necessary to prevent spontaneous activation of nociceptive neurons. To that end, we gradually reduced the $g_{\text{Kv}7/\text{M}}^{\text{Terminal}}$ and examined at which $g_{\text{Kv}7/\text{M}}^{\text{Terminal}}$ the simulated neuron starts to fire spontaneously (Figure 5). We found that even after inhibition of 63% of $g_{\text{Kv}7/\text{M}}^{\text{Terminal}}$ the remaining $g_{\text{Kv}7/\text{M}}^{\text{Terminal}}$ is sufficient to prevent spontaneous firing (Figure 5A). Lowering $g_{\text{Kv}7/\text{M}}^{\text{Terminal}}$ beyond 37% elicited spontaneous firing with a frequency which progressively increases with decrease of $g_{\text{Kv}7/\text{M}}^{\text{Terminal}}$ (Figures 5A,B).

### Decrease in terminal K_v_7 conductance leads to increased responsiveness of the simulated nociceptive neuron

**Figure 6:**
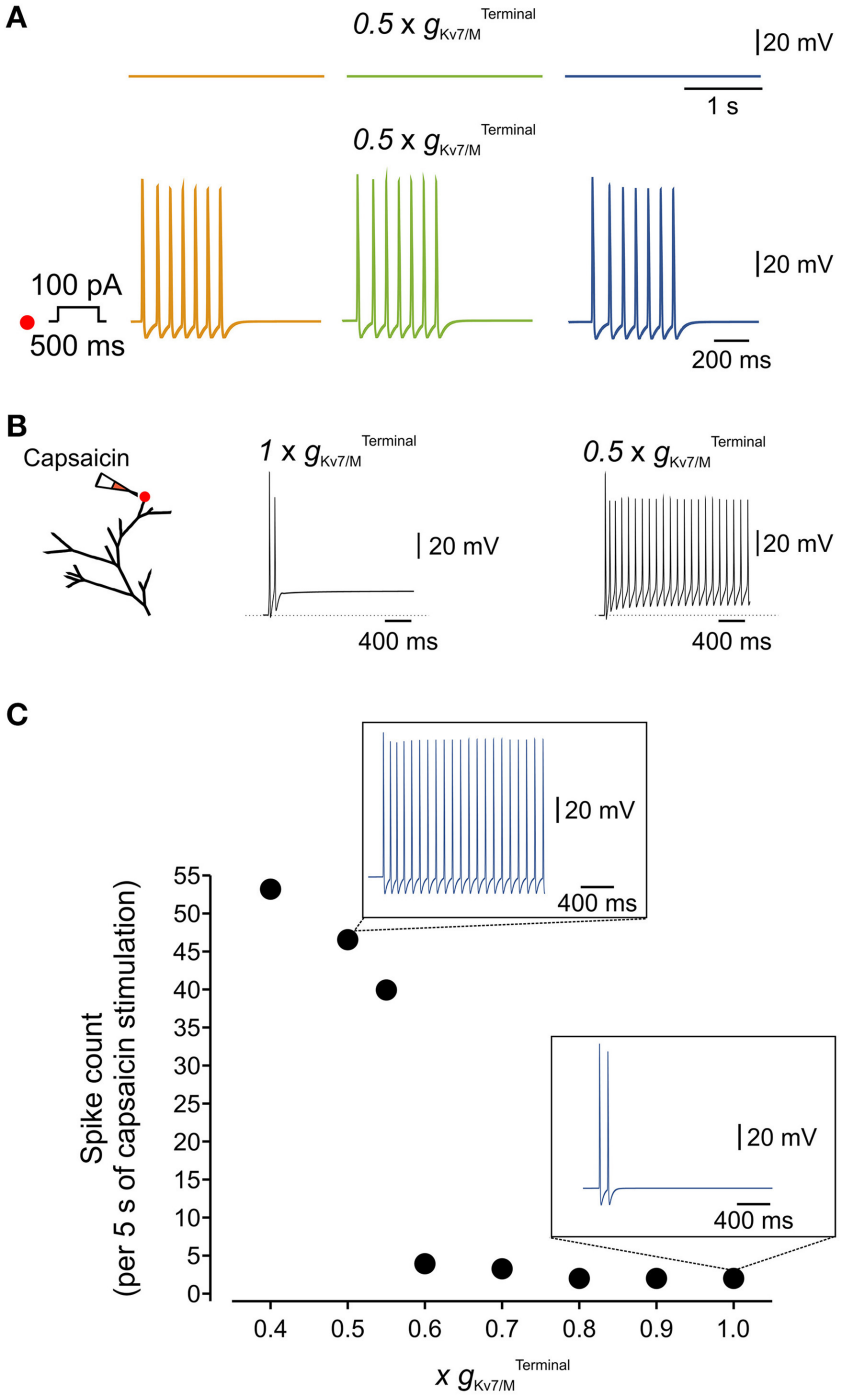
Terminal *I*_*M*_ controls the responsiveness of the simulated nociceptive neuron to incoming stimuli. **(A)** Blockade of 50% of terminal K_v_7/M channels did not lead to spontaneous activation of the simulated neuron (*upper traces*) but increased its responsiveness to a 100 pA, 500 ms current pulse applied to its distal terminal branch (*lower traces*, compare to Figure 4B). The location of stimulation is the same as in Figure 4. **(B)** Responses to application of capsaicin-like current. *Left*, Scheme of the modeled terminal showing the location of capsaicin application. *Middle*, recording from the simulated terminal following application of capsaicin-like stimulus when $g_{\text{Kv}7/\text{M}}^{\text{Terminal}}$ is intact. *Right*, the same stimuli induced a substantially stronger activation of the terminal when 50% of $g_{\text{Kv}7/\text{M}}^{\text{Terminal}}$ is inhibited. **(C)** Graph plotting changes in number of capsaicin-like stimulus' induced spikes (measured at the central axon during 5 sec application of capsaicin-like stimulus) with changes in $g_{\text{Kv}7/\text{M}}^{\text{Terminal}}$ (shown as a factor by which $g_{\text{Kv}7/\text{M}}^{\text{Terminal}}$ was multiplied). *Inset*s, Simulated recordings of changes in membrane potentials obtained from a multi-compartment model at the central axon (*blue electrode* in Figure 4) following the same capsaicin-like stimulus (shown in **(B)** when $g_{\text{Kv}7/\text{M}}^{\text{Terminal}}$ intact (*lower)* and reduced by half (*upper*). Note that decrease in $g_{\text{Kv}7/\text{M}}^{\text{Terminal}}$ elicited substantially stronger capsaicin-induced firing.

K_v_7/M channels are modulated by various signaling molecules. These channels require bound PIP_2_ for opening, and GqPCRs such as the muscarinic acetylcholine receptor causes *I*_M_ inhibition by PLC-induced PIP_2_ depletion (Suh and Hille, 2002; Zhang et al., 2003; Liu et al., 2010). Moreover, both IP_3_-induced increases in intracellular Ca^2+^ concentration (Jones et al., 1995) and PKC activation (Marrion, 1994; Lee et al., 2010) have been implicated in *I*_*M*_ inhibition. These signaling molecules and downstream signaling cascades are activated during inflammation. It is therefore plausible that during inflammation even partial inhibition of $g_{\text{Kv}7/\text{M}}^{\text{Terminal}}$ could increase the sensitivity of nociceptive neurons to incoming stimuli, thus leading to inflammatory hyperalgesia (Liu et al., 2010). We examined this hypothesis using our multi-compartment model in which we “inhibited” half of the $g_{\text{Kv}7/\text{M}}^{\text{Terminal}}\left(0.5\times g_{\text{Kv}7/\text{M}}^{\text{Terminal}}\right).$ Interestingly, although in these conditions, simulated neurons remained silent (Figure 6A, *upper traces*), stimulation of the terminal branch (as shown in Figure 6A, *right*, red dot) by a 500 ms square pulse of 100 pA current produced robust activation of the distal axon which propagated along the soma and reached the central terminal (compare Figure 6A, *lower traces* with Figure 4B, *lower traces*).

Next, we examined if partial inhibition of *I*_M_ would enhance the responsiveness of the simulated neuron to “natural” stimuli. We introduced the modeled TRPV1/capsaicin current (see Methods) into the terminals of the multi-compartment model. We applied a capsaicin-like stimulation onto the same terminal branch of the simulated neuron, previously used for electrical stimulation (Figure 6B, *left*, red dot), while varying the ${\text{g}}_{\text{Kv}7/\text{M}}^{\text{Terminal}}$. In “normal” conditions, i.e. with an intact $g_{\text{Kv}7/\text{M}}^{\text{Terminal}}$, application of the capsaicin-like stimulus induced relatively weak activation of the terminal (Figure 6B, *middle*) which was transmitted toward the central axon (Figure 6C, *lower inset*). We next examined whether inhibition of $g_{\text{Kv}7/\text{M}}^{\text{Terminal}},$ to levels which are not sufficient to induce spontaneous firing, would be sufficient to affect capsaicin-mediated responses of the multi-compartment model neuron. Inhibition of up to 30% of $g_{\text{Kv}7/\text{M}}^{\text{Terminal}}$ did not change the response of the terminal to capsaicin (*not shown*) and capsaicin-induced firing (Figure 6C). Further decrease of $g_{\text{Kv}7/\text{M}}^{\text{Terminal}}$ lead to a gradual increase in response to capsaicin-like stimulus (Figure 6C). Inhibition of more than 40% of $g_{\text{Kv}7/\text{M}}^{\text{Terminal}}$ induced robust, step-like increase in activation of the terminal (exemplified for ${0.5\;\times \;g}_{\text{Kv}7/\text{M}}^{\text{Terminal}}$ in Figure 6B, *right*), translated into about 11 Hz firing which was fully propagated toward the central terminal of the simulated neuron (Figure 6C, exemplified for ${0.5\times g}_{\text{Kv}7/\text{M}}^{\text{Terminal}}$ in Figure 6C, *upper inset)*.

### Terminal K_v_7 conductance stabilizes the membrane potential of modeled nociceptive terminals

**Figure 7:**
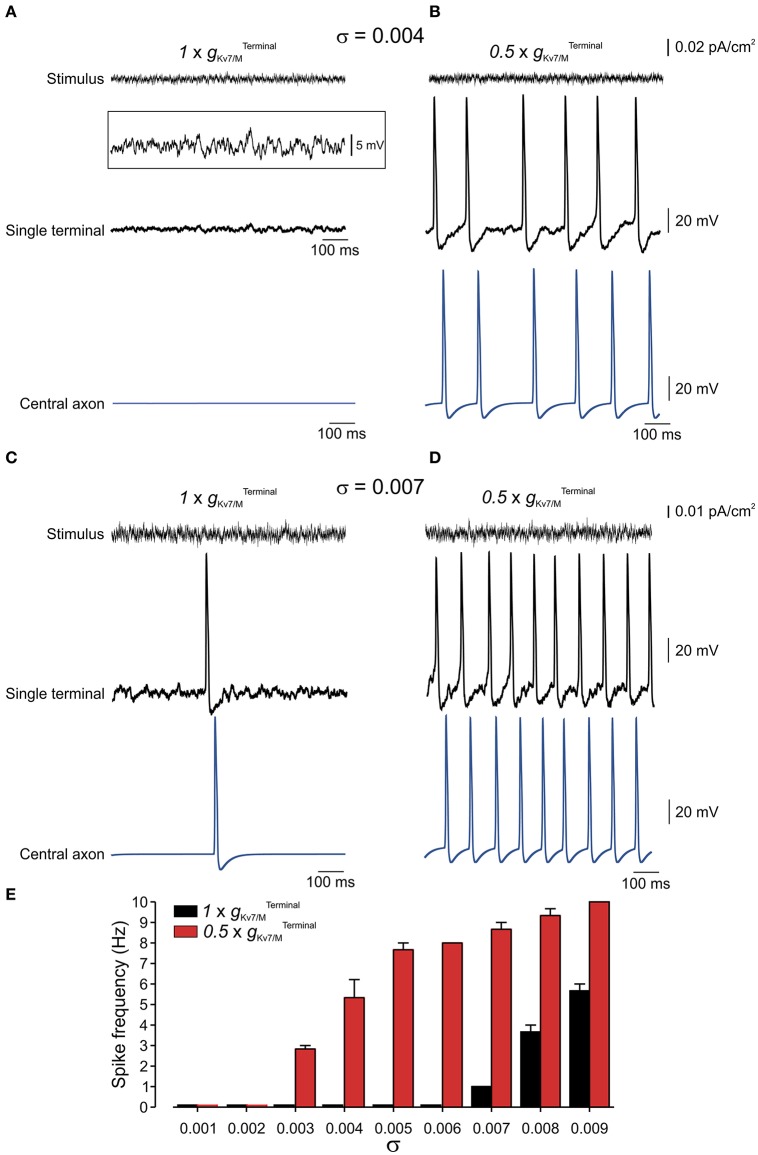
Terminal *g*_Kv7/M_ opposes membrane potential fluctuations. **(A)** *Upper*, Example of current noise trace (σ = 0.004) injected into the whole terminal tree of the multi-compartment model of the nociceptive neuron with intact ${\text{g}}_{\text{Kv}7/\text{M}}^{\text{Terminal}}.$ *Middle*, simulated recordings of changes in membrane voltage in single terminal following injection of the current noise (shown above), expanded in y-axis in *inset*. *Lower*, simulated recordings of changes in membrane voltage in central axon (*blue electrode* in Figure 4) following injection of the current noise (shown above) to the terminal tree. Note that at this noise level no activity is elicited at either terminal or soma. **(B)** Same as **(A)** but ${\text{g}}_{\text{Kv}7/\text{M}}^{\text{Terminal}}$ is reduced by half $\left(0.5\text{ x }{\text{g}}_{\text{Kv}7/\text{M}}^{\text{Terminal}}\right)$. **(C,D)** Same as **(A,B)** but with σ = 0.007. **(E)** Graph plotting changes in spike frequency with σ of current noise when ${\text{g}}_{\text{Kv}7/\text{M}}^{\text{Terminal}}$ intact (*black*) and reduced by half (*red*). Each bar represents 3 trials with a random noise input (see Methods). Note, increase in spike frequency as ${\text{g}}_{\text{Kv}7/\text{M}}^{\text{Terminal}}$ decreases.

Nociceptive membrane potential undergoes subthreshold fluctuating perturbations which are increased after nerve injury (Amir et al., 1999, 2002; Liu et al., 2000). To examine the contribution of *I*_*M*_ in impeding spontaneous firing during membrane fluctuations we have stimulated the terminal tree of the multi-compartment model by injecting an Onstein-Uhlenbeck-based current noise (see Methods), in which the current amplitude varies normally in each time-step of 0.25 ms (Olivares et al., 2015), while changing $g_{\text{Kv}7/\text{M}}^{\text{Terminal}}$. We varied the standard deviation of the current amplitude's normal distribution (σ) thus changing the probability of reaching a higher injected currents and causing higher membrane fluctuation amplitude (Figures 7A–D, *upper traces*). In normal conditions, i.e. when $g_{\text{Kv}7/\text{M}}^{\text{Terminal}}$ was intact, the modeled neuron remained quiescent despite fast membrane fluctuations at the single terminal of up to 15 mV (up to σ = 0.007; exemplified for σ = 0.004 in Figure 7A; exemplified for σ = 0.006 in Supplementary Figure 3; Figure 7F, *black*). When $g_{\text{Kv}7/\text{M}}^{\text{Terminal}}$ was reduced to half (Figure 7B), which, as we showed above, was not sufficient to induce spontaneous firing of the neuron (Figures 5, 6) neuronal membrane fluctuations of even 6 mV (σ = 0.004), were sufficient to elicit repetitive firing at the terminal tree which propagated toward the central terminal (Figures 7B,E, *red*).

In suprathreshold fluctuations, in which σ was increased to evoke low frequency firing (exemplified for σ = 0.007 in Figure 7C) reduction of $g_{\text{Kv}7/\text{M}}^{\text{Terminal}}$ to half led to a substantial increase in firing frequency of nociceptive neurons (Figures 7D,E). These data predict that *I*_*M*_ reduces susceptibility of nociceptive neurons to membrane fluctuations. When $g_{\text{Kv}7/\text{M}}^{\text{Terminal}}$ is decreased the nociceptive neuron is activated by small perturbations of 6 mV, while when $g_{\text{Kv}7/\text{M}}^{\text{Terminal}}$ is intact, the neurons will still be quiescent even upon 15 mV fluctuations.

### Terminal K_v_7 conductance affects nociceptive firing in pathological conditions

**Figure 8:**
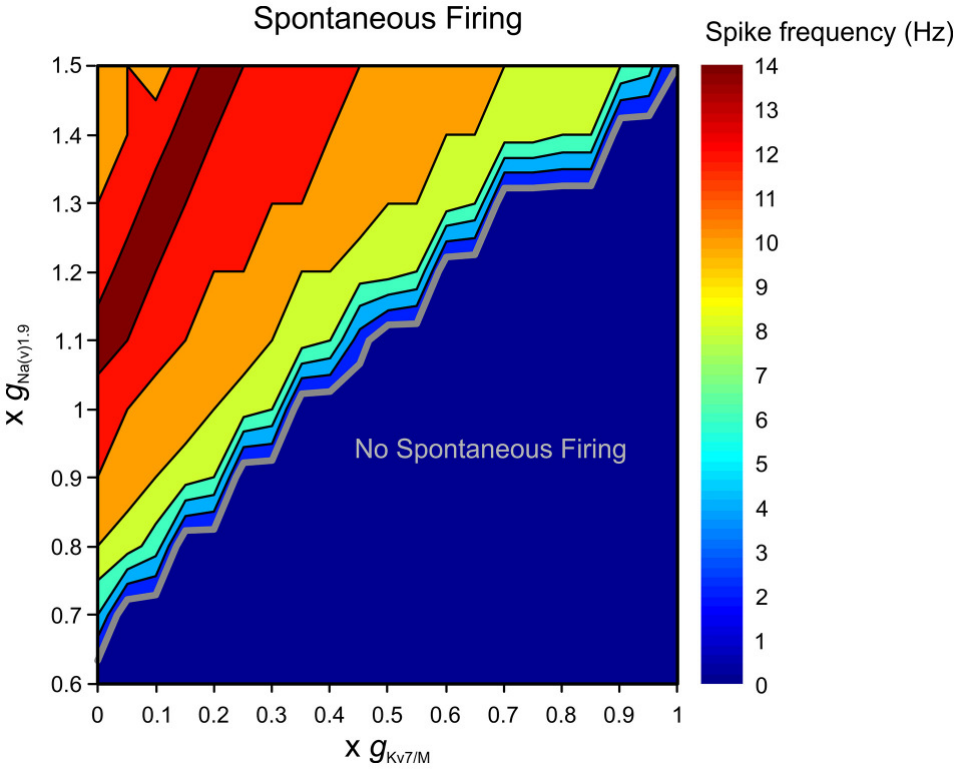
Terminal *g*_Kv7/M_ opposes *g*_Na(v)1.9_ in preventing spontaneous firing. Graphic representation of the relationship between the terminal *g*_Kv7_ and *g*_Na(v)1.9_ in eliciting spontaneous firing of multi-compartmental model of nociceptive neurons. The recordings performed at the proximal axon of the modeled neuron (*blue electrode* in Figure 4). The thick gray line outlines the range of *g*_Kv7_ and *g*_Na(v)1.9_ beneath which no spontaneous activity occurs (*blue area*). The frequency of the spontaneous activity is color coded (shown on the *right*). Note that when $g_{\text{Kv}7/\text{M}}^{\text{Terminal}}$ is intact, spontaneous firing can be elicited only when *g*_Na(v)1.9_ is increased above 140%. Note also that when $g_{\text{Kv}7/\text{M}}^{\text{Terminal}}$ is annulled, at least 65% of *g*_Na(v)1.9_ is required to induce spontaneous firing.

Subthreshold excitability of nociceptive neurons is controlled, among other factors, by depolarizing conductances, contributed largely by sodium channel isoform 1.9 (Na_(V)_1.9) which underlie persistent TTX-resistant sodium current (Herzog et al., 2001; Dib-Hajj et al., 2002; Baker, 2005). To characterize the interaction between $g_{\text{Kv}7/\text{M}}^{\text{Terminal}}$ and terminal *g*_Na(v)1.9_, we simulated, using our multi-compartmental model, the effect of changing these conductances on the excitability of a modeled nociceptive terminal and whole neuron. When $g_{\text{Kv}7/\text{M}}^{\text{Terminal}}$ was zeroed neurons remained silent if *g*_Na(v)1.9_ was inhibited (Figure 8). In our conditions, at least 65% of terminal *g*_Na(v)1.9_ were required for spontaneous firing when $g_{\text{Kv}7/\text{M}}^{\text{Terminal}}$ was zeroed. These data suggest that at subthreshold potentials *I*_*M*_ counteracts TTX-resistant persistent sodium current, thus precluding spontaneous firing.

The enhancement of *g*_Na(v)1.9_ under pathological conditions has been shown to lead to activation of nociceptive neurons (Baker, 2005; Amaya et al., 2006; Binshtok et al., 2008; Maingret et al., 2008; Gudes et al., 2015). We examined the ability of ${\text{g}}_{\text{Kv}7/\text{M}}^{\text{Terminal}}$ to prevent spontaneous activity when *g*_Na(v)1.9_ was enhanced. We show that when $g_{\text{Kv}7/\text{M}}^{\text{Terminal}}$ is intact the simulated neuron remains silent even when *g*_Na(v)1.9_ is increased up to 145% (Figure 8). *I*_*M*_ was also able to prevent stimulus-induced hyper-responsiveness when *g*_Na(v)1.9_ was increased up to 125% (Supplementary Figure 4). On the other hand, when $g_{\text{Kv}7/\text{M}}^{\text{Terminal}}$ was decreased, even a slight increase in *g*_Na(v)1.9_ resulted in spontaneous firing, such that when $g_{\text{Kv}7/\text{M}}^{\text{Terminal}}$ was reduced by half, about 10% increase in *g*_Na(v)1.9_ produced activation of modeled nociceptive neuron (Figure 8). As stated before, in these conditions, stimulus-evoked hyper-responsiveness was present even when *g*_Na(v)1.9_ was intact (see Figure 6A, Supplementary Figure 4).

Altogether these data suggest that when terminal *I*_M_ is intact no spontaneous activity will occur even during substantial perturbation in depolarizing conductances in subthreshold range. It also implies that in pathological conditions spontaneous activation of nociceptive neurons could be more easily achieved by the cooperative effect of decrease in *g*_Kv7_ and increase in *g*_Na(v)1.9_.

## Discussion

Here we show that in peripheral nociceptive neurons *I*_*M*_ plays a predominant role in stabilizing membrane potential at subthreshold potentials, acting to prevent ectopic activity of nociceptive neurons, in addition to its part in regulating nociceptive responsiveness to stimuli. We showed that inhibition of *I*_M_, in addition to increasing the susceptibility of nociceptive neurons to applied stimuli, also leads to spontaneous firing. Using a realistic multi-compartment computational model of peripheral nociceptive neurons we show that not all K_v_7/M channels conductance at the nociceptive terminal tree is required to prevent spontaneous firing, but only ~40% of it is sufficient for this matter. We demonstrated that the “spare” K_v_7/M conductance may serve to provide a “safety zone” against depolarizing membrane perturbations and to some extent to negate increasing subthreshold depolarizing conductances.

Activation of *I*_*M*_ underlies a slow noninactivating K^+^ current known to be generated by K_V_7 channel subunits. In nociceptive neurons K_V_7.2/3 (KCNQ2/3) are predominately responsible for providing the majority of *I*_*M*_ (Mucha et al., 2010; Du and Gamper, 2013). We and others have demonstrated that in nociceptive DRG neurons, these channels are activated at near resting potential such that at subthreshold potentials they produce a prominent outward current (Figure 2; see also Brown and Adams, 1980; Passmore et al., 2003), helping to keep the resting potential at hyperpolarized range. In addition to *I*_*M*_, the subthreshold excitability of DRG neurons is controlled by other, albeit weaker outward conductances (K2P and 4-AP sensitive voltage gated potassium channels, Du et al., 2014) which are counteracted by inward conductances: the persistent sodium current mediated largely by the Na(v)1.9 subtype of voltage gated sodium channels (Herzog et al., 2001) and hyperpolarization-activated cyclic nucleotide-gated *I*_h_ mediated mainly by HCN2 (Emery et al., 2011). Enhancement of Na_(V)_1.9-mediated current and activation of *I*_h_ has been shown to produce firing of DRG neurons (Baker et al., 2003; Baker, 2005; Emery et al., 2011), hence positioning active *I*_*M*_ as a major factor to restrain membrane depolarization (Du et al., 2014) and prevent neuronal activation in lack of depolarizing stimuli. We show here, using pharmacological means, that *I*_*M*_-mediated conductance alone is sufficient to silence the nociceptive neurons. This conclusion is also supported by studies showing that application of the K_v_7/M channels enhancers retigabine (Main et al., 2000) or NH6 (Peretz et al., 2007) lead to membrane hyperpolarization and reduces excitability of nociceptive DRG neurons (Passmore et al., 2003; Rivera-Arconada and Lopez-Garcia, 2006; Peretz et al., 2007). Moreover, retigabine reduces neuropathy-induced nocifencive behavior (Blackburn-Munro and Jensen, 2003) and prevents spontaneous firing of nociceptive fibers in models of neuropathic pain (Roza and Lopez-Garcia, 2008; Bernal et al., 2016).

Several recent reports demonstrated that inhibition of *I*_*M*_ lead to increased responsiveness of nociceptor-like DRG neurons toward depolarizing stimuli (Passmore et al., 2003; Mucha et al., 2010; Shi et al., 2013; Du et al., 2014). *I*_*M*_ has been shown to act as a brake on repetitive firing of nociceptive neurons such that its inhibition increases the firing rate of nociceptive neurons upon application of suprathreshold currents (Passmore et al., 2003). Moreover, inhibition of *I*_*M*_ decreases action potential threshold together with inducing mild depolarization (Passmore et al., 2003; Linley et al., 2008). These effects together, if sufficient, could potentially lead to generation of spontaneous firing. Interestingly, although XE991-mediated spontaneous firing was reported in CA1 neurons and in spinal cord motor neurons (Shah et al., 2008; Lombardo and Harrington, 2016; Tzour et al., 2016), no XE991-mediated spontaneous firing was shown for nociceptive DRG neurons. It was suggested that XE991-induced depolarization increases the inactive fraction of sodium channels, probably via a slow inactivation mechanism (Blair and Bean, 2003; Binshtok et al., 2008; Gudes et al., 2015), which precludes action potential firing (Linley et al., 2008). If the latter is true, the differences in XE991 application could explain this discrepancy. In our experiments we used focal puff application of XE991. It could very well be that rapid access of relatively homogeneous concentrations of XE991 lead to depolarization with faster kinetics than the kinetics of sodium channels slow inactivation, rendering enough available sodium channels for action potential generation.

Kv7.2 channels are expressed both by nociceptive DRG somata and by their nerve endings (Passmore et al., 2003, 2012). XE991 injected subcutaneously at the rat's hind paw, induced nocifencive behavior (Linley et al., 2008), implying that inhibition of *I*_M_ is sufficient to activate nociceptive terminals. To casually link between inhibition of *I*_*M*_ at the soma and terminals and the firing of nociceptive neurons we have built computational models of nociceptive somata and of a whole nociceptive neuron. While building this in-silico nociceptive neuron we used data from the experimental results, when available, but for some of the parameters some assumptions had to be made. For example, since there are no available experimental data describing the various ion channel conductances at the terminal and along the axon, we assumed an equal and homogeneous distribution of the conductances between the soma, terminal and axon. We show here that lowering of terminal *g*_Kv7/M_, i.e. decrease in terminal Kv7/M channels density, lead to substantial increase in nociceptive excitability. If, on the other hand, the density of the Kv7/M channels at the terminals is higher than that of the soma it will widen the “safety zone” provided by Kv7/M channels. However, other factors such as spatial distribution of Kv7/M channels and their co-localization with other channels (Battefeld et al., 2014) may affect the impact of Kv7/M channels on nociceptive excitability. In our model, for simplicity, we used a uniform distribution of all ion conductances including that of the Kv7/M channels along the terminal and axon. A recent study on cortical neurons shows that in their axon initial segment and nodes of Ranvier, Kv7/M channels are co-localized with the sodium channels (Battefeld et al., 2014). In these nodal domains, Kv7/M channels, by stabilizing the membrane resting potential, increase the availability of sodium channels, thus enhancing neuronal excitability. In the somato-dendritic domain of the cortical neurons, where Kv7/M channels are present in lower density and no co-localization with sodium channels was observed, activation of Kv7/M channels dampen neuronal excitability (Battefeld et al., 2014). It has been shown that application of retigabine onto nociceptive terminals *in vivo* inhibits evoked neuronal activity, whereas application of XE991 enhances it (Passmore et al., 2012), suggesting that Kv7/M channels in the nociceptive terminal tree play a restricting role on nociceptive excitability. However, it is plausible that further along the peripheral axon, where sodium channels are arranged in higher density lipid rafts (Pristera et al., 2012), or in central terminals of the nociceptive axon, Kv7/M channels are not equally distributed but co-localized with the sodium channels. In this case they may affect nociceptive excitability differently form their effect on the terminal tree or the cell body. Therefore, although our models replicate well the experimental results (Figure 3), we make no claim that it models all nociceptive neurons in all states. The purpose of our models was to show the plausibility of our interpretation of sufficiency of *g*_Kv7/M_ to prevent ectopic activation of nociceptive neurons. Indeed, our single and multi-compartment model predicts the effect of inhibition of somatic or terminal K_v_7/M conductance by showing spontaneous firing of the simulated neuron when K_v_7/M conductance has been inhibited at the soma or terminal tree (Figures 3, 4). Interestingly, our simulated data suggest that in order to remove the *I*_*M*_-mediated clamping from the soma of DRG-like neurons almost all (98%) K_v_7/M conductance has to be inhibited while in the terminals, spontaneous firing could be induced by inhibiting 63% of K_v_7/M conductance. Recent results using an immunofluorescent approach have demonstrated that in neuropathic pain models the expression level of K_v_7.2 in soma of injured neurons decreased by 65% (Cisneros et al., 2015). According to our model such a decrease may not be enough to produce spontaneous firing at DRG somata, but may lead to increase of their responsiveness to applied stimuli. However, if a similar decrease in K_v_7 expression occurs homogenously along the neuron including the terminal tree, 65% decrease in K_v_7/M conductance could be sufficient to induce ectopic activation, which may underlie nerve-injury mediated spontaneous pain.

In yet another case of pathological pain - inflammation - proinflammatory mediators act on a variety of subthreshold conductances to increase nociceptive excitability thus leading to inflammatory pain (Baker, 2005; Amaya et al., 2006; Binshtok et al., 2008; Maingret et al., 2008; Emery et al., 2011; Gudes et al., 2015). We have shown here that *I*_*M*_, when intact, provides a “safety zone” which prevents spontaneous firing even when some of the subthreshold depolarizing conductances are substantially increased. However, K_v_7/M channels are by themselves common targets for proinflammatory mediators (Linley et al., 2008; Liu et al., 2010; Tzour et al., 2016). The proinflammatory agent, bradykinin was shown to decrease K_v_7/M conductance, albeit by less than 50% (Liu et al., 2010). Our results show that such decrease may not be sufficient to elicit spontaneous firing. However, in the inflammatory conditions bradykinin acting together with other ingredients of the inflammatory soup, also increase persistent sodium current (Maingret et al., 2008). This complex effect of inflammatory mediators on the one hand narrowing the *I*_*M*_-provided “safety zone” and on other hand increasing persistent sodium current could lead to ectopic activation of nociceptive neurons and to inflammatory pain.

Collectively, our data position *I*_*M*_ as a main factor acting to prevent spontaneous firing. It could however be that this is only true for specific subtypes of nociceptive neurons. It has been recently demonstrated that application of XE991 onto *ex-vivo* skin-nerve preparation lead to spontaneous activity in mechanical and heat sensitive Aδ fibers but not C-fibers (Passmore et al., 2012). This suggests that *I*_*M*_ is sufficient to silence nociceptive Aδ fibers similarly to what we have shown here. It also implies that at sensory endings of C-fibers other conductances also contribute to stable membrane potential at the subthreshold levels (see Du et al., 2014).

In conclusion, our findings in combination with those of previous studies (see Delmas and Brown, 2005) suggest that K_v_7/M channels at nociceptive neurons, are critical for controlling resting membrane potential hence impeding ectopic activity and pathological pain. Our data show that in normal conditions, *I*_*M*_ provide a “safety zone” sufficient to oppose substantial depolarizatory deviations of membrane potentials thus preventing the membrane to reach action potential threshold. Finally, our data demonstrated that spontaneous firing of nociceptive neurons in pathological conditions strongly depends on a decrease in *g*_K_v_7/M_, emphasizing the importance of pharmacological enhancement of *g*_K_v_7/M_ in treatment of pathological pain.

## Author contributions

OB: Conceived the study, designed and conducted the experiments, built the model, analyzed the data and wrote the manuscript; RG and YC: Conducted the experiments, analyzed the data and wrote the manuscript; BK and SL: Analyzed the data and wrote the manuscript; AB: Conceived the study, designed the experiments, analyzed the data, supervised the project and wrote the manuscript.

### Conflict of interest statement

The authors declare that the research was conducted in the absence of any commercial or financial relationships that could be construed as a potential conflict of interest. The handling Editor declared a shared affiliation, though no other collaboration, with the authors and states that the process nevertheless met the standards of a fair and objective review.

## Supplementary material

Supplementary Figure 1 *I*_*M*_ is blocked by 3 μM XE991 in nociceptor-like DRG neurons. **(A)** Voltage-clamp perforated patch recordings from a nociceptor-like DRG neuron using the protocol to isolate *I*_*M*_ (see Methods and legend of Figure 2) before and 10 min after application of 3 μM XE991. Each subpanel illustrates a family of currents evoked by a series of 1 s, 5 mV hyperpolarizing voltage steps from a holding potential of −20 mV (voltage protocol is shown in *inset*). The dashed line indicates zero current level. Note, a decrease in the holding current. The peak current responses obtained by stepping to −50 mV are shown at the bottom of each subpanel. The red line is a bi-exponential fit of *I*_*M*_. The dotted line indicates the levels of steady state currents before command offset. Note XE991 inhibition of *I*_*M*_ (representative of 4/4 experiments). **(B)** Subtracted trace of peak current response obtained before (shown in **(A)**, *bottom left*) from those after application of XE991 (shown in **(A)**, *bottom right*). Lines as in **(A)**. **(C)** Bar graph comparing peak *I*_*M*_ amplitudes measured at −50 mV (showed in **(B)**, *inset*) in the two conditions as in **(A)**; ^***^*p* < 0.001, Student *t*-test; *n* = 4.

Supplementary Figure 2 Capsaicin-induced current and voltage responses in nociceptor-like DRG neurons. **(A)** Representative trace of a capsaicin-induced current (1 μM) recorded evoked by 5 s puff application (representative of 10 neurons). **(B)** Representative trace of the changes in membrane potential in nociceptor-like neuron shows substantial depolarization followed by action potential discharge (*inset*: expanded time scale) induced by puff application of capsaicin (5 s, 1 μM). The dotted line indicates the membranes potential before the stimulation (−58 mV, representative of 10 neurons).

Supplementary Figure 3 Terminal *g*_Kv7/M_ opposes membrane potential fluctuations. **(A)** *Upper*, Example of a current noise trace (σ = 0.006) injected into the whole terminal tree of the multi-compartment model of nociceptive neuron with intact $g_{\text{Kv}7/\text{M}}^{\text{Terminal}}$. *Middle*, simulated recordings of changes in membrane voltage in single terminal following injection of the current noise (showed above), expanded in y-axis in *inset*. *Lower*, simulated recordings of changes in membrane voltage in the central axon (*blue electrode* in Figure 4) following injection of the current noise (showed above) to the terminal tree. Note that at this noise level no activity is elicited at either terminal or soma. **(B)** Same as (A) but $g_{\text{Kv}7/\text{M}}^{\text{Terminal}}$ is reduced by half. Note that in this conditions same noise level elicited spontaneous firing.

Supplementary Figure 4 The limits of terminal *g*_Kv7/M_ in preventing stimulus induced hyper-responsiveness of modeled nociceptive neuron. Graphic representation of the relationship between the terminal *g*_*Kv*7_ and *g*_Na(v)1.9_ in eliciting increased responsiveness to 500 ms, 100 pA square pulses. In normal conditions, when $g_{\text{Kv}7/\text{M}}^{\text{Terminal}}$ = $1\text{ x}g_{\text{Kv}7/\text{M}}^{\text{Terminal}}$ and *g*_Na(v)1.9_ = 1 x *g*_Na(v)1.9_, this stimuli evokes 1 spike (*blue area*, see also Figure 4B). The recordings performed at the proximal axon of the modeled neuron (*blue electrode* in Figure 4). The number of spikes during 500 ms step color coded (shown on the right). Note that when ${\text{g}}_{\text{Kv}7/\text{M}}^{\text{Terminal}}=1\text{ x }g_{\text{Kv}7/\text{M}}^{\text{Terminal}}$, the number of spikes is increased when *g*_Na(v)1.9_ is increased above 120%.
