# Supplementary figures and images for: The Role of Kv7/M Potassium Channels in Controlling Ectopic Firing in Nociceptors
Source: Front Mol Neurosci. 2017 Jun 13;10:181. doi: 10.3389/fnmol.2017.00181 (PMC5468463; doi:10.3389/fnmol.2017.00181)

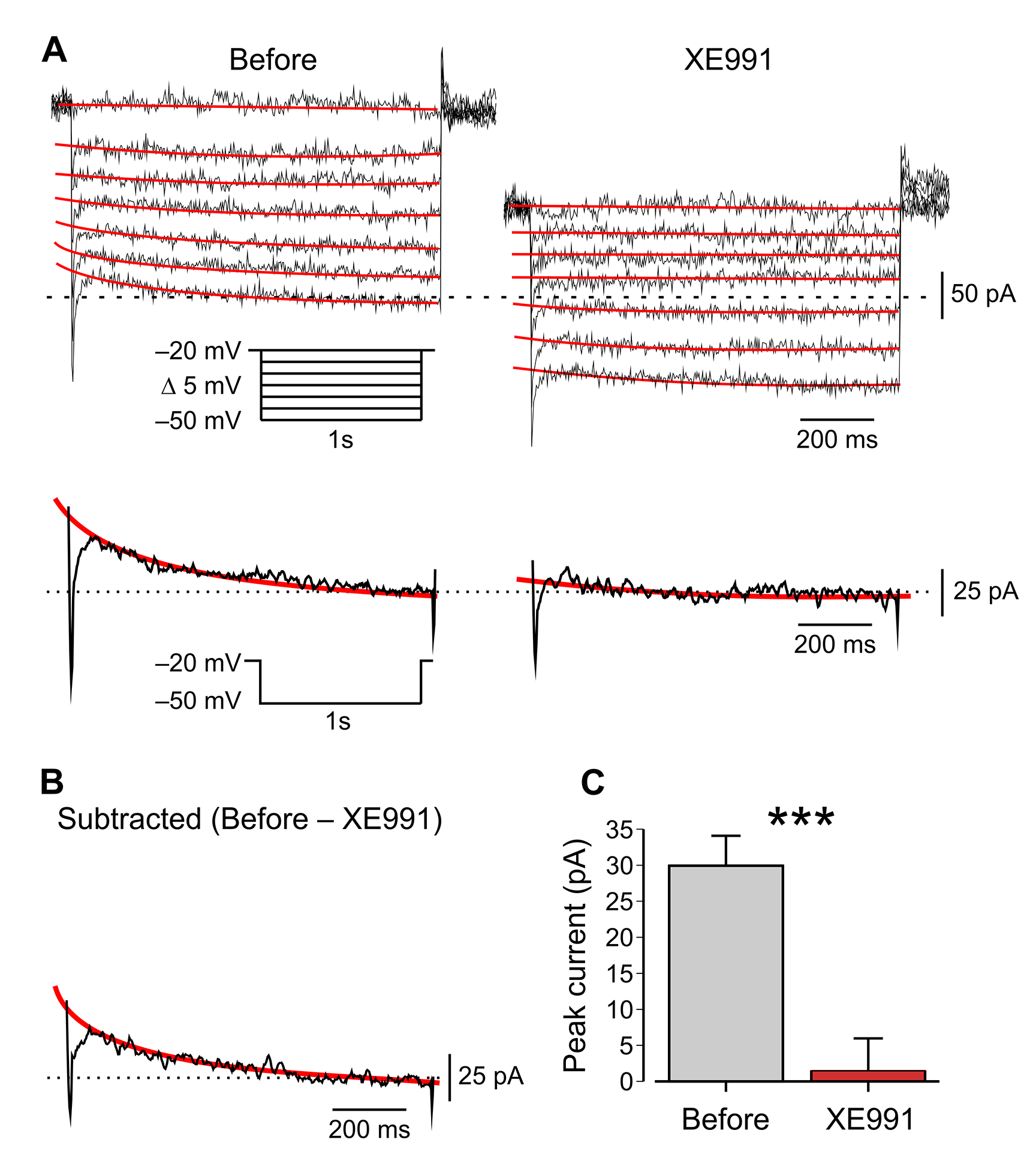

Supplement: Supplementary Figure 1 — IM is blocked by 3 μM XE991 in nociceptor-like DRG neurons. (A) Voltage-clamp perforated patch recordings from a nociceptor-like DRG neuron using the protocol to isolate IM (see Methods and legend of Figure 2) before and 10 min after application of 3 μM XE991. Each subpanel illustrates a family of currents evoked by a series of 1 s, 5 mV hyperpolarizing voltage steps from a holding potential of −20 mV (voltage protocol is shown in inset). The dashed line indicates zero current level. Note, a decrease in the holding current. The peak current responses obtained by stepping to −50 mV are shown at the bottom of each subpanel. The red line is a bi-exponential fit of IM. The dotted line indicates the levels of steady state currents before command offset. Note XE991 inhibition of IM (representative of 4/4 experiments). (B) Subtracted trace of peak current response obtained before (shown in (A), bottom left) from those after application of XE991 (shown in (A), bottom right). Lines as in (A). (C) Bar graph comparing peak IM amplitudes measured at −50 mV (showed in (B), inset) in the two conditions as in (A); ***p < 0.001, Student t-test; n = 4. [file Image1.TIF]

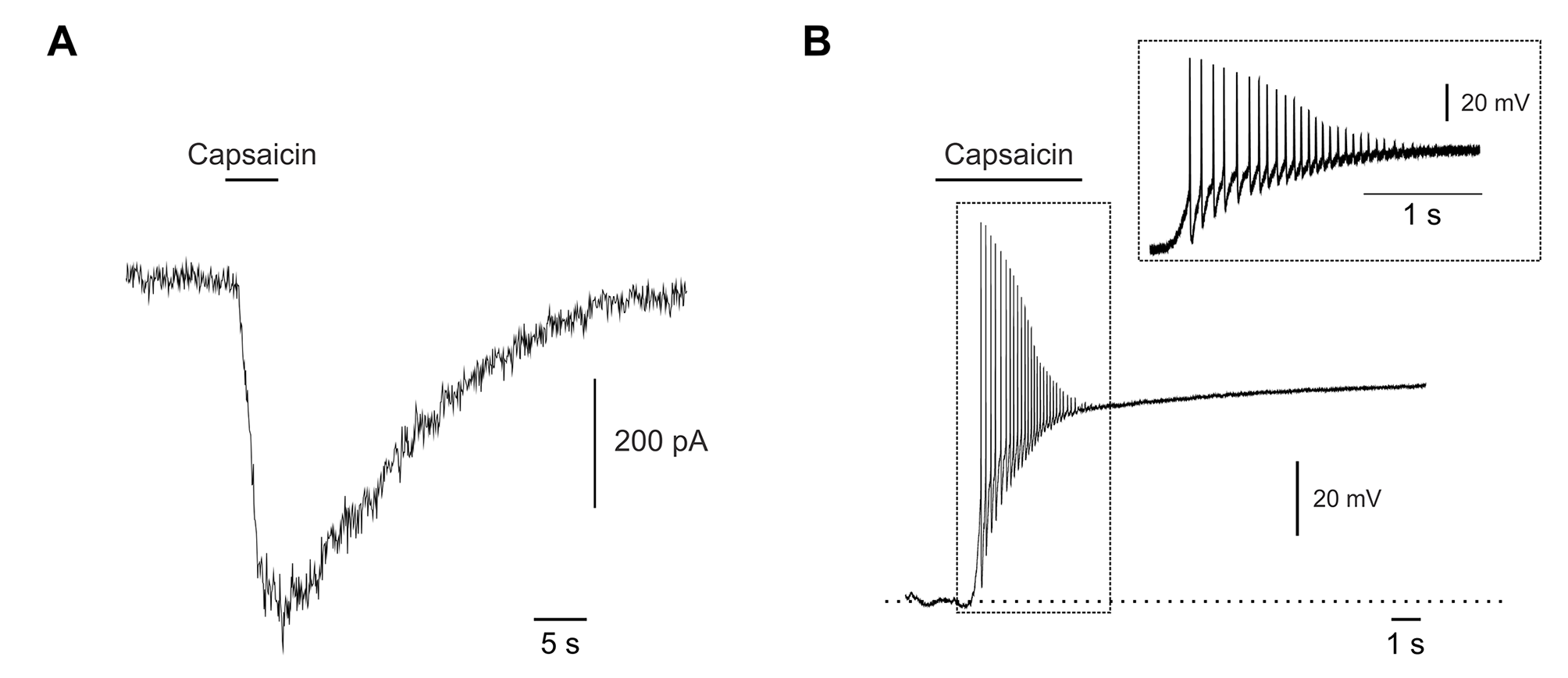

Supplement: Supplementary Figure 2 — Capsaicin-induced current and voltage responses in nociceptor-like DRG neurons. (A) Representative trace of a capsaicin-induced current (1 μM) recorded evoked by 5 s puff application (representative of 10 neurons). (B) Representative trace of the changes in membrane potential in nociceptor-like neuron shows substantial depolarization followed by action potential discharge (inset: expanded time scale) induced by puff application of capsaicin (5 s, 1 μM). The dotted line indicates the membranes potential before the stimulation (−58 mV, representative of 10 neurons). [file Image2.TIF]

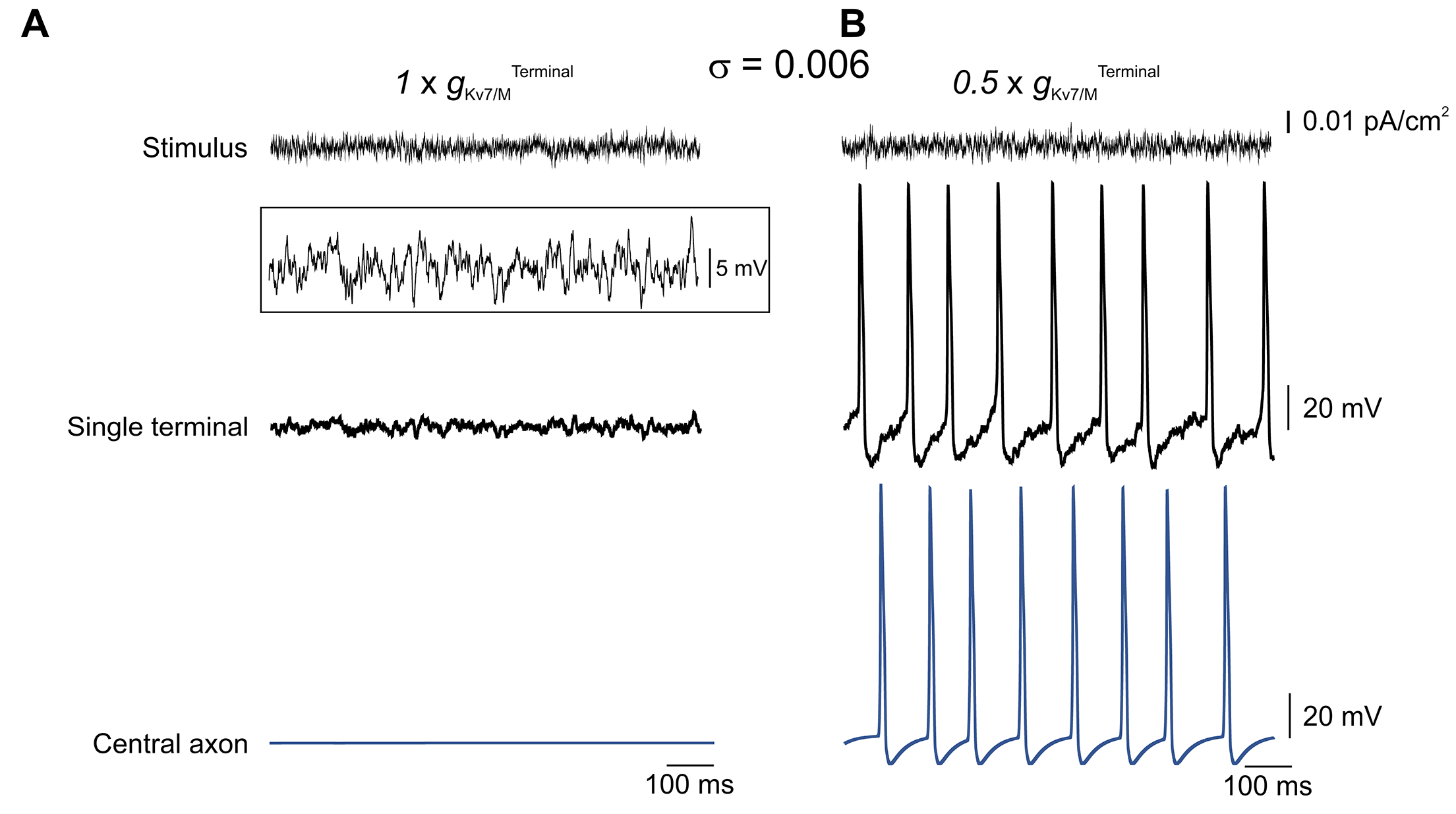

Supplement: Supplementary Figure 3 — Terminal gKv7/M opposes membrane potential fluctuations. (A) Upper, Example of a current noise trace (σ = 0.006) injected into the whole terminal tree of the multi-compartment model of nociceptive neuron with intact gKv7/MTerminal. Middle, simulated recordings of changes in membrane voltage in single terminal following injection of the current noise (showed above), expanded in y-axis in inset. Lower, simulated recordings of changes in membrane voltage in the central axon (blue electrode in Figure 4) following injection of the current noise (showed above) to the terminal tree. Note that at this noise level no activity is elicited at either terminal or soma. (B) Same as (A) but gKv7/MTerminal is reduced by half. Note that in this conditions same noise level elicited spontaneous firing. [file Image3.TIF]

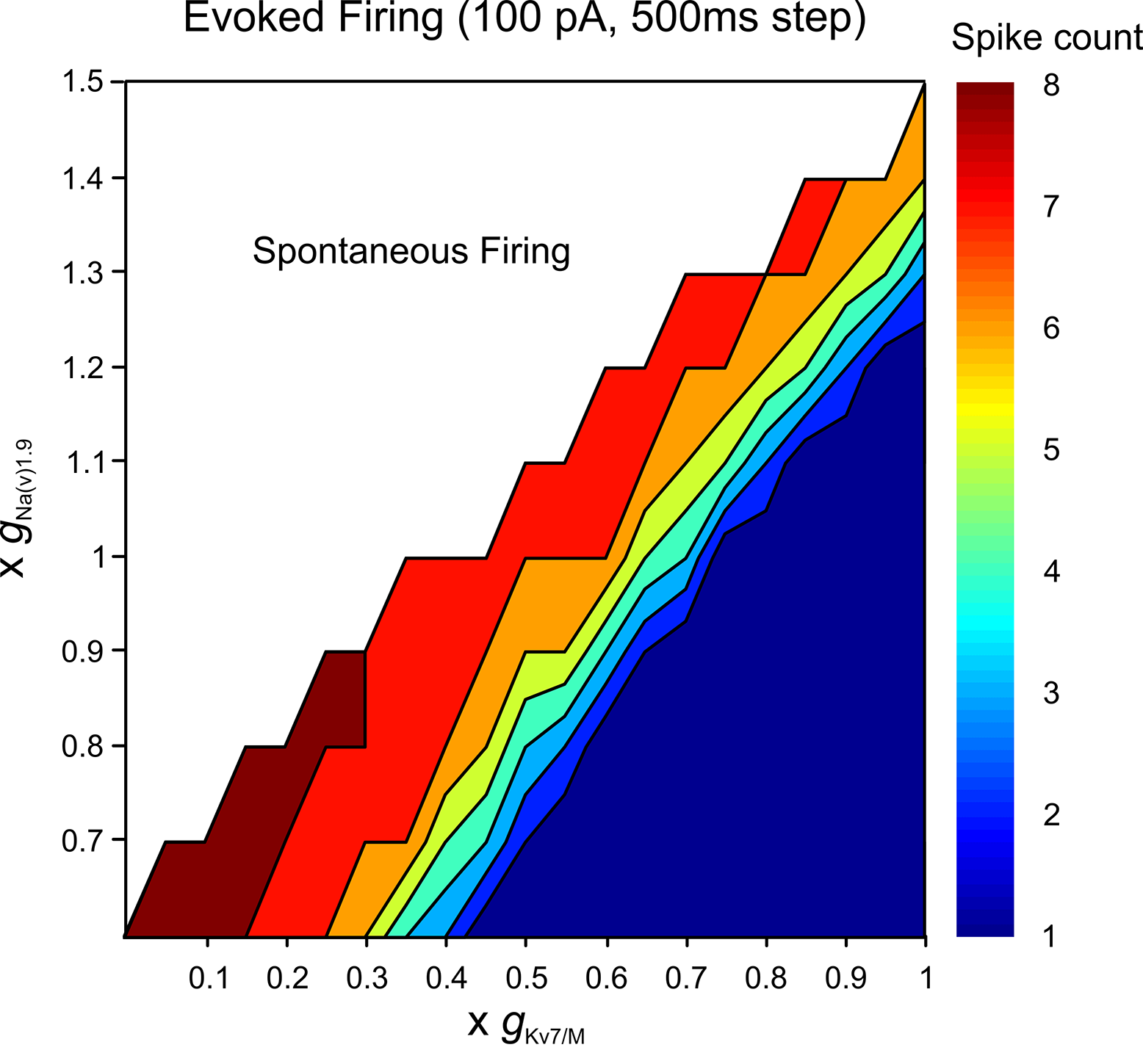

Supplement: Supplementary Figure 4 — The limits of terminal gKv7/M in preventing stimulus induced hyper-responsiveness of modeled nociceptive neuron. Graphic representation of the relationship between the terminal gKv7 and gNa(v)1.9 in eliciting increased responsiveness to 500 ms, 100 pA square pulses. In normal conditions, when gKv7/MTerminal = 1 xgKv7/MTerminal and gNa(v)1.9 = 1 x gNa(v)1.9, this stimuli evokes 1 spike (blue area, see also Figure 4B). The recordings performed at the proximal axon of the modeled neuron (blue electrode in Figure 4). The number of spikes during 500 ms step color coded (shown on the right). Note that when gKv7/MTerminal=1 x gKv7/MTerminal, the number of spikes is increased when gNa(v)1.9 is increased above 120%. [file Image4.TIF]
